# Detection of Immune Checkpoint Receptors – A Current Challenge in Clinical Flow Cytometry

**DOI:** 10.3389/fimmu.2021.694055

**Published:** 2021-07-01

**Authors:** Benjamin Shibru, Katharina Fey, Stephan Fricke, André-René Blaudszun, Friederike Fürst, Max Weise, Sabine Seiffert, Maria Katharina Weyh, Ulrike Köhl, Ulrich Sack, Andreas Boldt

**Affiliations:** ^1^ Institute of Clinical Immunology, Medical Faculty, University of Leipzig, Leipzig, Germany; ^2^ Fraunhofer Institute for Cell Therapy and Immunology (IZI), Leipzig, Germany; ^3^ Institute for Cellular Therapeutics, Hannover Medical School, Hannover, Germany

**Keywords:** checkpoint receptors, immune diagnostics, flow cytometry, immune oncology, infection, immunity, autoimmunity, laboratory diagnose

## Abstract

Immunological therapy principles are increasingly determining modern medicine. They are used to treat diseases of the immune system, for tumors, but also for infections, neurological diseases, and many others. Most of these therapies base on antibodies, but small molecules, soluble receptors or cells and modified cells are also used. The development of immune checkpoint inhibitors is amazingly fast. T-cell directed antibody therapies against PD-1 or CTLA-4 are already firmly established in the clinic. Further targets are constantly being added and it is becoming increasingly clear that their expression is not only relevant on T cells. Furthermore, we do not yet have any experience with the long-term systemic effects of the treatment. Flow cytometry can be used for diagnosis, monitoring, and detection of side effects. In this review, we focus on checkpoint molecules as target molecules and functional markers of cells of the innate and acquired immune system. However, for most of the interesting and potentially relevant parameters, there are still no test kits suitable for routine use. Here we give an overview of the detection of checkpoint molecules on immune cells in the peripheral blood and show examples of a possible design of antibody panels.

## Introduction

In recent years, medical diagnostic laboratories have witnessed dynamic changes in the field of cellular immunodiagnostics.

Those are based on several factors such as i) improvements of flow cytometers and their software, which allows multi-parameter diagnostics with 12 and more colors even for routine laboratories, ii) deepened immunological findings, which suggest a pathogenetic relevance for numerous parameters, and iii) a variety of new therapies, which directly or indirectly affect the immune system. Those changes must be described in order to optimally care for those patients.

Normally, only “Conformité Européenne” (CE)-labeled *in-vitro* diagnostic medical devices (IVD) are used in patient diagnostics (1). However, due to the high dynamics in this field, the large number of antibodies, the required flexibility in the composition of combinations, and the different characteristics of the available laboratory equipment, it is not possible to use test kits to any significant extent.

Here, we will provide an overview about checkpoint molecules with diagnostic potential. This is not a complete list, but we have limited ourselves to molecules for which reliable publications are available and for which diagnostic relevance is suspected. Although the expression of checkpoint molecules on T cells is the focus of many studies, these markers can be detected on virtually all cells of the innate and acquired immune system. Therefore, we present exemplary cell populations expressing these molecules.

In order to flexibly respond to the challenges of this fast-growing number of immune markers, we set up a combination of antibodies in our laboratory that can be flexibly combined with additional markers. We show examples for several cell populations which markers we can detect this way. We know that these protocols are not provided as IVD and must be set up thoroughly. This is a challenge in clinical practice (2). For validation, recent publications give support (3). Reference values are often not known and must be established in-house (4). We present how we analyze them in a specialized routine laboratory and give examples for T-cells, monocytes, NK cells, and PMNs.

**Figure 1:**
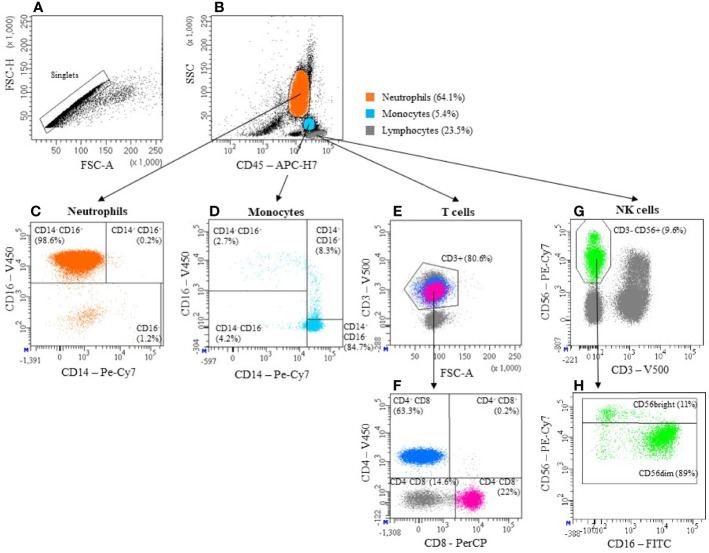
Gating strategy. The basis of all measurements in this publication is the gating strategy shown here. After exclusion of doublets **(A)**, Neutrophils, Monocytes and Lymphocytes were identified based on the expression of CD45 and granularity (SSC) **(B)**. Neutrophils are also defined by high CD16 and low CD14 expression (CD14-CD16+) **(C)**. Monocytes can be categorized into 3 subpopulations, based on their expression pattern of CD14 and CD16: i) “classical” CD14+CD16-, ii) “intermediate” CD14+CD16+ and iii) “non-classical” CD14-CD16+ **(D)**. T cells were defined as Lymphocytes expressing CD3 **(E)**. By confronting CD4 and CD8 we then identified cytotoxic T cells (CD4^-^ CD8^+^) and T helper cells (CD4^+^ CD8^-^) **(F)**. Among Lymphocytes, those cells that express CD56 but not CD3 were defined as NK cells **(G)**. They were further divided into a CD56dim (CD56^+^) and a CD56bright (CD56^++^) subset **(H)**. Antibody panels used can be found in **Table 1**.

All examinations were performed in an accredited immunological laboratory according to the International Standard DIN EN ISO 15189:2012 (5). The flow cytometric measurement gave us a general overview of the distribution of peripheral blood cells (**Figure 1**). Antibodies applied in our investigation are listed in **Table 1**. For each sample, 100 µl of whole blood was incubated with an antibody cocktail specific for the desired cell populations. After surface cell staining for 15 min at room temperature in the dark, erythrocytes were lysed by incubation with lysis buffer (BD Biosciences, Heidelberg, Germany) for 10 min. Lymphocytes were then fixed with 200 µl PBS (Biochrom, Berlin, Germany) containing 1% formaldehyde.

**Table 1:** Panel description and specification of antigens, fluorochromes, clones, distributors, and quantity of antibodies used for staining of 100 µl whole blood.

Antigen	Fluorochrome	Clone	Company	µl Antibody/100 µl blood
*Panel (i): T cell 1*				
CD3	V500	UCHT1	BD Biosciences	2,5
CD45RA	PerCP-Cy5.5	HI100	eBioscience	5
CD197 (CCR7)	BV421	2-L1-A	BD Horizon	2,5
TIGIT	Alexa Fluor 647	A15153G	BioLegend	5
PD-1	PE	EH12.1	BD Pharmingen	5
*Panel (ii): T cell 2*				
CD4	V450	RPA-T4	BD Horizon	2,5
CD8	PerCP	SK1	BD Horizon	5
CD45	APC-H7	2D1	BD Pharmingen	2,5
TIGIT	Alexa Fluor 647	A15153G	BioLegend	5
PD-1	PE	EH12.1	BD Pharmingen	5
*Panel (iii): T cell 3*				
CD4	V450	RPA-T4	BD Horizon	2,5
CD8	PerCP	SK1	BD Horizon	5
CD45	APC-H7	2D1	BD Pharmingen	2,5
CD3	V500	UCHT1	BD Horizon	2,5
PD-1	PE	EH12.1	BD Pharmingen	5
Tim-3	APC	F38-2E2	BioLegend	5
Lag-3	FITC	11C6C65	BioLegend	5
BTLA	PE-Cy7	MIH26	BioLegend	5
*Panel (iv): NK cell 1*				
CD3	V500	UCHT1	BD Bioscience	2,5
CD16	FITC	3G8	BioLegend	2,5
CD45	APC-H7	2D1	BD Pharmingen	2,5
CD56	PE-Cy 7	NCAM16.2	BD Bioscience	2,5
Lag-3	PE	11C3C65	BioLegend	5
Tim-3	APC	F38-2E2	BioLegend	5
*Panel (v): NK cell 2*				
CD3	V500	UCHT1	BD Bioscience	2,5
CD16	FITC	3G8	BioLegend	2,5
CD45	APC-H7	2D1	BD Pharmingen	2,5
CD56	PE-Cy7	NCAM16.2	BD Bioscience	2,5
Siglec-7	PE	6-434	BioLegend	5
TIGIT	Alexa Fluor 647	A15153G	BioLegend	5
*Panel (vi): Monocyte 1*				
CD45	APC-H7	2D1	BD Pharmingen	2,5
CD16	V450	3G8	BD Horizon	2,5
CD14	Pe-Cy7	M5E2	BD Pharmingen	2,5
HLA-DR	PerCP	L243	BD Bioscience	5
SIRPα	FITC	15-414	BioLegend	5
Tim-3	APC	F38-2E2	BioLegend	5
LILRB2	PE	42D1	BioLegend	5
*Panel (vii): Monocyte 2*				
CD45	APC-H7	2D1	BD Pharmingen	2,5
CD16	V450	3G8	BD Horizon	2,5
CD14	Pe-Cy7	M5E2	BD Pharmingen	2,5
HLA-DR	PerCP	L243	BD Bioscience	5
TIGIT	Alexa Flour 647	A15153G	BioLegend	5
VISTA	PE	MIH65.rMAb	BD Pharmingen	5
LILRB4	BV510	ZM3.8	BDOptiBuild	2,5
*Panel (vii): Monocyte 3*				
CD45	APC-H7	2D1	BD Pharmingen	2,5
CD16	V450	3G8	BD Horizon	2,5
CD14	Pe-Cy7	M5E2	BD Pharmingen	2,5
HLA-DR	PerCP	L243	BD Bioscience	5
PD-1	PE	EH12.1	BD Pharmingen	5
*Panel (viii): Neutrophil 1*				
CD45	PerCP	2D1	BioLegend	5
CD16	V450	3G8	BD Horizon	2,5
CD14	PE-Cy7	M5E2	BD Pharmingen	2,5
PD-1	FITC	MIH4	BD Pharmingen	5
VISTA	PE	MIH65.rMAb	BD Pharmingen	5
Tim-3	APC	F38-2E2	BioLegend	5
*Panel (ix): Neutrophil 2*				
CD45	PerCP	2D1	BioLegend	5
CD16	V450	3G8	BD Horizon	2,5
CD14	PE-Cy7	M5E2	BD Pharmingen	2,5
SIRPα	FITC	15-414	BioLegend	5
LILRB2	PE	42D1	BioLegend	5
TIGIT	Alexa Fluor 647	A15153G	BioLegend	5

For data acquisition, an eight color FACS Canto II flow cytometer (BD Biosciences) was used, equipped with a 405 nm violet laser, a 488 nm blue laser and a 647 nm red laser. All the data were analyzed using FACS DIVA (BD Biosciences) software. The expression of checkpoint molecules was given in relative values (percentages).

Finally, we give examples of checkpoint regulation in human pathologies, focusing on tumors, infection, and autoimmunity. Here, we refrain from a comprehensive presentation of PD-1 and CTLA-4 on T cells, as a broad body of data already exists in this area.

## Immune Cells Relevant in Checkpoint Detection

### T-Cells

T-cells derive from hematopoietic stem cells. Through several processes of maturation, there are different subpopulations that differ not only in their function within the immune system but also in expression of unique markers. T-cells express CD3 and the T-cell-receptor (TCR), as well as CD4 or CD8 (6). When considering T-cells, these both molecules will be focused on in this paper, as the detection of CD4 as well as CD8 on the cell surface is suitable to reliably identify T-cells through flow cytometry (**Figure 1**). We hereby state that essential T-cell subpopulations are not selectively detected in this way.

T-cell activation as well as survival and expansion are achieved through three main signals: i) interaction of TCR with antigen peptide-loaded major histocompatibility complex I or II (MHC-I/II) on antigen-presenting cells (APC), ii) interaction of CD28 on T-cells with CD80 (B7-1) expressed on APC or CD86 (B7-2) found on B-cells and monocytes, which results in a co-stimulatory signal (7) and iii) cytokines secreted by APCs that direct differentiation into T cell subsets.

Beyond that, several immune checkpoints interact with signaling pathways in T-cell activation. Immune checkpoints gained huge interest as they indicate and finally offer an opportunity to modulate the effectiveness of the human immune system. Long time established therapies to tumors or chronic diseases are often limited by severe adverse events as they come with drastic interference with the immune system. Immune checkpoints expressed on T-cells are therefore subject to many studies aiming at establishing an inhibitor. In this paper there we focus on TIGIT, LAG-3, TIM-3, PD-1, and BTLA as some common examples of immune checkpoints.

### NK-Cells

Natural Killer (NK) cells are part of a heterogenous group called innate lymphoid cells (ILCs). Even though they derive from common CD34^+^ lymphoid progenitors, they do not express a genetically rearranged antigen receptor (8). Because NK cells uniquely express CD56 but neither CD19 nor CD3, common markers of B- and T-cells respectively, they can be easily identified using flow cytometry.

Accounting for 10-15% of all lymphocytes, NK cells can be further differentiated into two main subsets, based on the expression levels of CD56 and CD16 (9) (**Figure 1**). The immature CD56^bright^ CD16^+/-^ subset is predominantly localized in tissue and secondary lymphoid organs and produces cytokines (IFN-γ, TNF-α, GM-CSF) and chemokines (CCL2, CCL3, CCL4, CCL5). The fully mature CD56^dim^ CD16^+^ subpopulation accounts for 90% of NK cells in the peripheral blood and possesses a potent cytotoxic capacity. However, contrary to earlier believes, those main effector functions cannot be unambiguously split up between the subsets. CD56^dim^ NK cells contribute significantly to early cytokine production (10) and both CD56^dim^ and CD56^bright^/CD16^+^ and CD16^-^ change during cytokine stimulation (11).

NK cells kill their targets by releasing lytic granules that contain Granzymes, Perforin, Fas ligand (FasL, CD178), TNF-related apoptosis-inducing ligand (TRAIL, CD253), Granulysin and small anti-microbial peptides (12). Activity of NK cells is determined by a homeostasis of germline encoded activating and inhibitory receptors. The Natural Cytotoxicity Receptors (NCRs): NKp30, NKp44 and NKp46 as well as activating forms of KIR, 2B4 and NKG2D are some of the activating receptors expressed on NK cells. Furthermore, FcγRIIIA facilitates antibody-dependent cellular cytotoxicity (ADCC), through its ability to recognize IgG opsonized targets. While most of those activating receptors recognize ligands that are expressed by abnormal cells, many inhibitory receptors like inhibitory KIRs and CD94/NKG2A recognize classical or non-classical MHC-I molecules as signs of self. Cells under stress often change the expression of ligands for those activating or inhibitory receptors and thus the homeostasis may shift towards activation of the NK cells (12, 13).

For example, it is common for tumors and virus infected cells to escape immunosurveillance by cytotoxic T-cells through a loss of MHC-I and thus NK cells close a gap that is left by adaptive immunity (13).

**Figure 2:**
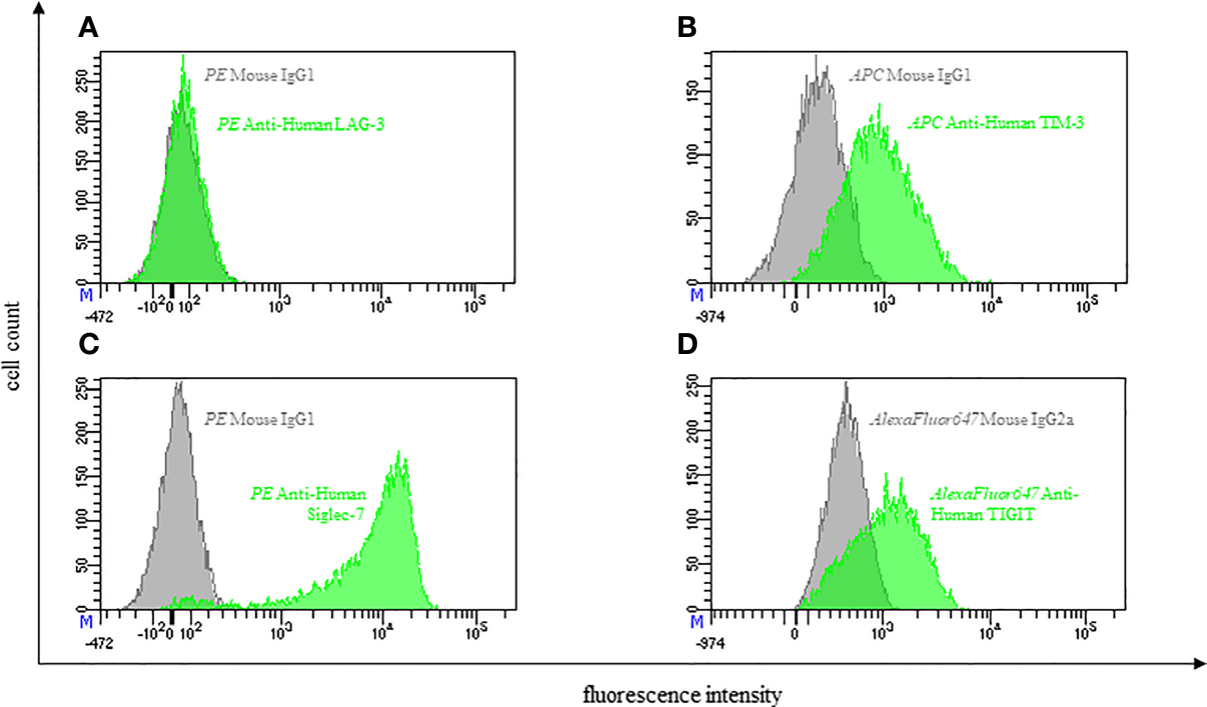
Representative flow cytometric analysis of the expression of immune checkpoints (green): LAG-3 **(A)**, TIM-3 **(B)**, Siglec-7 **(C)** and TIGIT **(D)** on resting NK cells of a healthy donor (male, 23 years old) compared with isotype control (grey).

Based on work in our lab, this review will focus on TIM-3, LAG-3, TIGIT and SIGLEC-7 as representatives of immune checkpoints on NK cells (**Figure 2**). This selection is by no means a complete representation of all immune checkpoints expressed on NK cells.

### B-Cells

B-cells are antigen presenting cells (APCs) which form the cellular source of antibodies (14, 15). Stimulation of the B-cell receptor (BCR) with its cognate antigen initiates a cascade of intracellular signaling, leading to internalization of that antigen for processing and presentation in context of major histocompatibility complex class II molecules (MHC-II) to the T-cell receptor (TCR) of CD4^+^ T-cells (16–19). By interaction of the antigen peptide/MHC-II complex and the TCR, the CD4^+^ T-cell gets activated and secretes cytokines leading to an antibody class switch of the B-cell. Consequently, the activated B-cell differentiates into a plasma cell, which produces and secretes soluble antibodies against the matching antigen (17, 20, 21). In addition to antigen presentation and antibody production, activated B cells are also able to generate immunological memory cells and carry out regulatory functions (15, 22–25).

B-cells carry checkpoint ligands on theirs surface including PD1-L, CD80/CD86 and ICOS-L (26–28). They also express CD40 (CD154), a member of the tumor necrosis factor receptor superfamily. Its ligand CD40-L is classically expressed on CD4^+^ T-cells (29).

CD40 is a transmembrane protein acting as a signal transducer, which activates intracellular kinases and transcription factors as well as the production of antibodies and a variety of cytokines. Moreover, it influences apoptosis and regulates expression of surface molecules (30). Clearly, the CD40/CD40-L pathway is the most potent activator of B-cells (31, 32). It is also known that the CD40/CD40-L pathway regulates the costimulatory activity of B-cells, this directly influences T-cell activation (22, 33, 34).

In the past few years several therapeutic strategies, especially in treatment of autoimmune disease, such as rheumatoid arthritis, and lymphomas have been developed including targeting surface markers like CD20 with Rituximab and by disrupting inter- or intracellular functions, for example targeting CD40-L with Toralizumab or Ruplizumab (35–40).

Tumor-infiltrating B-cells have been identified, but their precise functional role in the tumor microenvironment (TME) is still unclear. In some studies, it was demonstrated that B-cells are tumor-promoting, others suggest that there is a positive association with improved cancer outcomes, especially when they are found in association with tertiary lymphoid structures (TISs) (41–43). In absence of requests, we not yet included B-cells in our diagnostic panels.

### Monocytes

Monocytes are a subgroup of leukocytes, belonging to the innate immune system. Deriving from a myeloid progenitor cell in the bone marrow, they circulate in the blood to detect any kind of pathogens. They are able to enter tissues where they differentiate into macrophages. Depending on what stimuli they encounter, they can either differentiate into M1 or M2 macrophages. M1 macrophages are considered to promote inflammation by producing proinflammatory cytokines. M2 macrophages have a different function as they regulate and inhibit immune response by producing anti-inflammatory cytokines (44). These different macrophage phenotypes play an important role in cancer. Current studies analyze how tumor derived extracellular vesicles (EV) are able to modulate monocyte-derived macrophages phenotype and cytokine profile (45). Some studies suggest that these EVs contribute to M2 polarization and thereby promote tumor immune evasion and tumor growth (46).

Monocytes detect pathogens with their pattern recognition receptors. Identified pathogens are phagocytized, internalized, and processed into antigen fragments in a phagolysosome. These fragments activate T-cells when presented *via* MHC II receptors. Besides detection of pathogens, phagocytosis and antigen presentation, monocytes also have a secretory function. They produce different anti- and pro-inflammatory cytokines to regulate inflammatory responses. Therefore, they also release chemokines to lure other immune cells to the inflammatory site. Other secretory products are complement factors and growth factors (47).

Monocytes can be divided into three groups according to their surface expression of CD14 and CD16: classical monocytes are CD14^++^CD16^-^, intermediate monocytes express both (CD14^+^CD16^+^) and non-classical monocytes express high levels of CD16 and low levels of CD14 (CD14^low^CD16^high^) (48)(**Figure 1**). Classical monocytes make up about 80-90% of all monocytes and promote inflammation. Intermediate monocytes account for 2-5% but show an increased proportion in several inflammatory conditions such as sepsis, various viral infection, and autoimmune diseases. 5-10% are supposed to be non-classical monocytes with a more anti-inflammatory phenotype (49).

**Figure 3:**
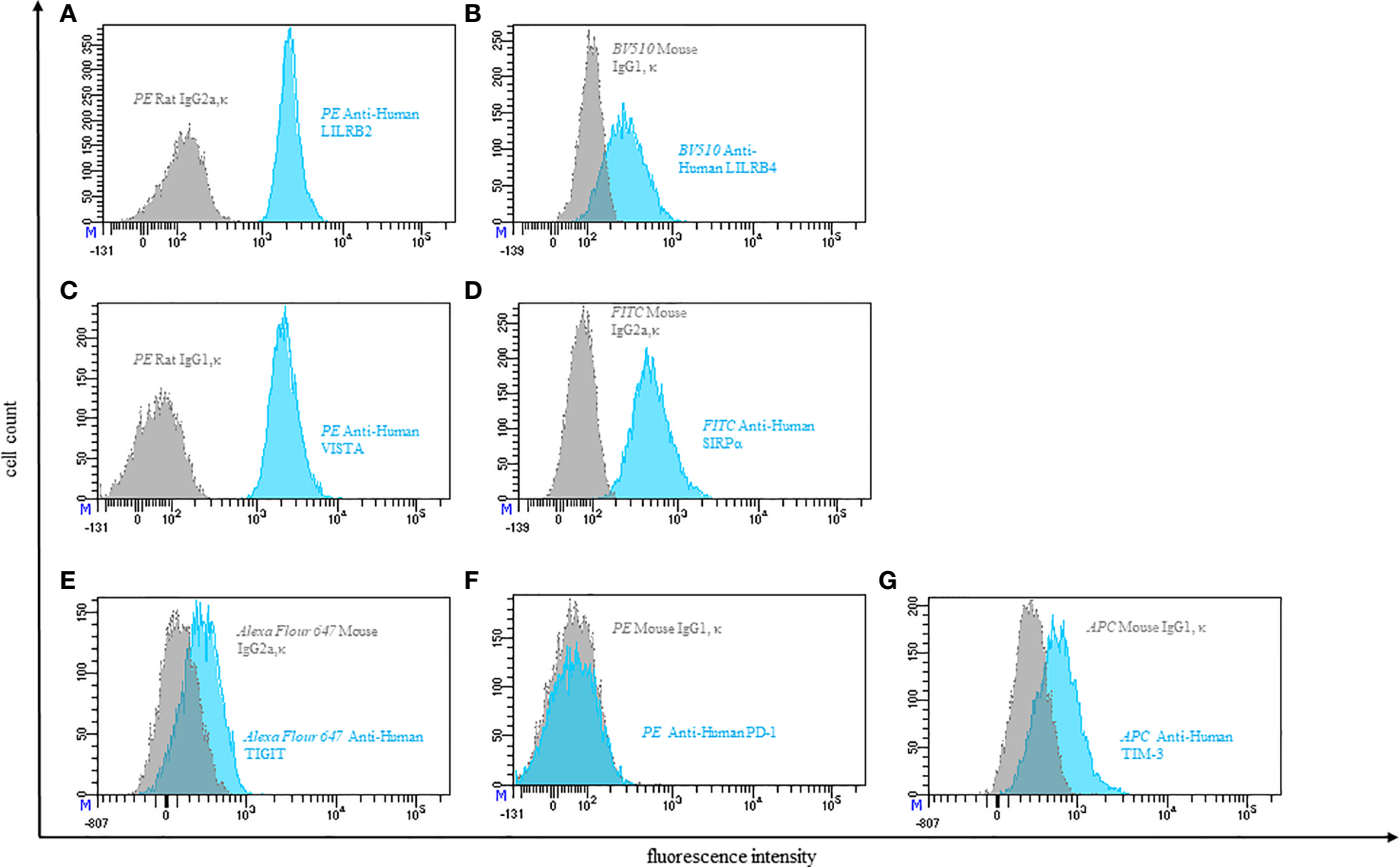
Representative flow cytometric analysis of the expression of immune checkpoints (blue): LILRB2 **(A)**, LILRB4 **(B)**, VISTA **(C)**, SIRPα **(D)**, TIGIT **(E)**, PD-1 **(F)**, TIM-3 **(G)** on resting peripheral blood monocytes of a healthy donor compared with isotype control (grey).

Monocytes are important in maintaining immune balance and inhibiting excessive immune responses. When expressing negative immune checkpoint receptors on their surface they downregulate immune responses due to reduced cytokine secretion or inhibition of immune responses of other immune cells when interacting with them. In order to offer an overview of common immune checkpoints expressed on monocytic surfaces this paper attends to SIRPα, TIM-3, PD-1, TIGIT, VISTA, LILRB2 and 4 (**Figure 3**).

### Neutrophils

Neutrophils play a major role in immune defense against microorganisms. They are the first cells to be recruited during acute inflammation and possess a variety of effector mechanisms to generate effective immune responses (50).

In addition, the importance of neutrophils in the tumor microenvironment (TME) has become increasingly clear over the last decade. Similar to tumor-associated macrophages (TAMs), tumor-associated neutrophils (TANs) can be subclassified into an anti-tumorigenic “N1” and a pro-tumorigenic “N2” phenotype in this context (51).

It is well established that within other cell populations of the immune system co-inhibitory and co-stimulatory stimuli generated by checkpoint molecules play a crucial role in regulating and adapting immune responses. The neutrophil response to invading pathogens must also be tightly controlled in order to avert excessive inflammation and tissue damage. However, it is not certain whether immune checkpoints participate in this regulation of neutrophil responses.

Studies have shown that neutrophils express several immune checkpoints such as PD-1 (52), VISTA (53, 54) and SIRPα (55) and Siglec-7 (56). However, functions and immunological relevance remain to be characterized. Only LILRB2 expression and function on human neutrophils has been further studied.

**Figure 4:**
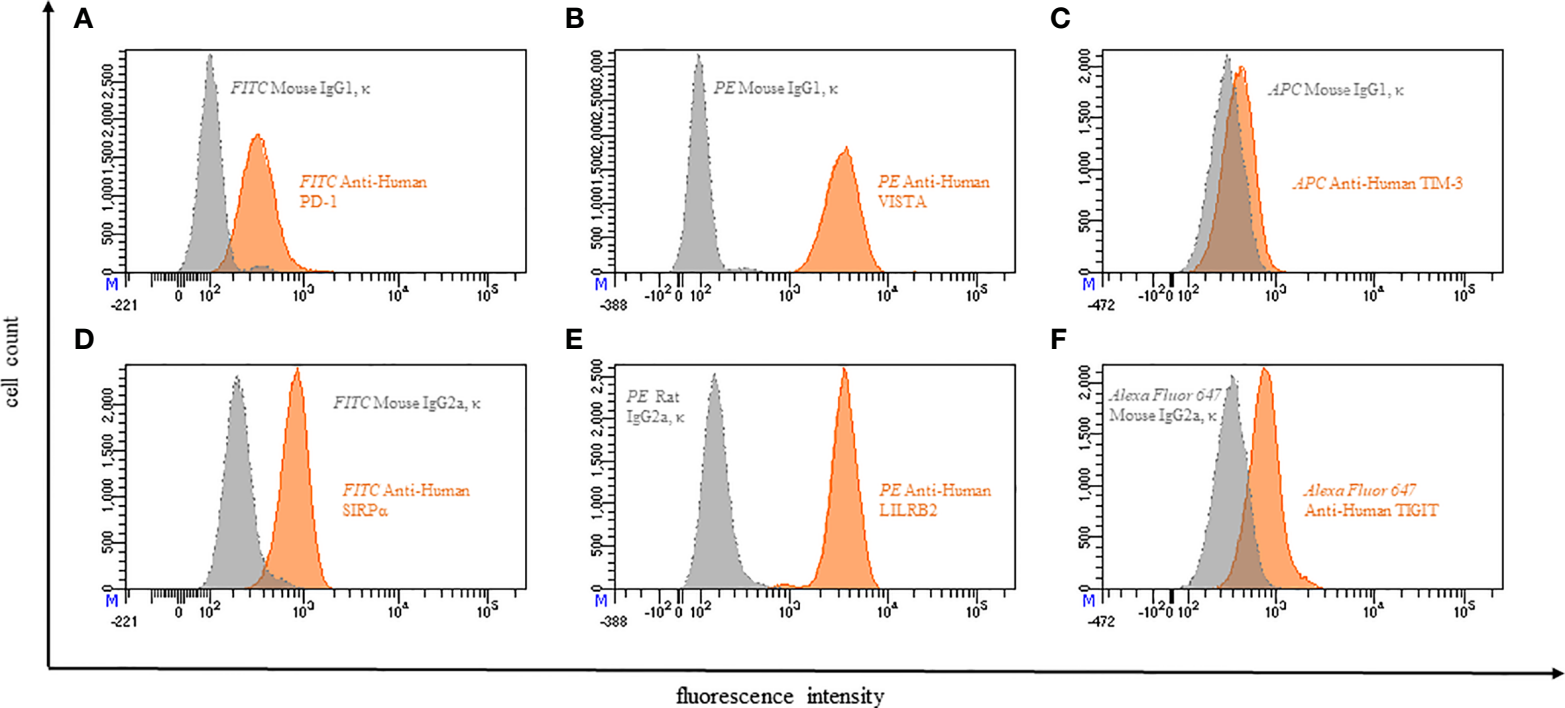
Representative flow cytometric analysis of the expression of immune checkpoints (orange): PD-1 **(A)**, VISTA **(B)**, TIM-3 **(C)**, SIRPα **(D)**, LILRB2 **(E)**, TIGIT **(F)** on resting neutrophils of a healthy donor compared with isotype control (grey).

In order to expand the knowledge of immune checkpoint expression on neutrophils, we analyzed the expression of PD-1, VISTA, TIM-3, TIGIT, SIRPα and LILRB2 on neutrophils (**Figure 4**).

### Dendritic Cells

Dendritic cells (DCs) are the most potent antigen presenting cells (APCs). They can be found in almost all tissues, where they play a central role in regulation of the adaptive immune response. DCs are uniquely able to induce primary naïve T-cell activation and effector differentiation (57, 58). In comparison to other cells in the immune system, their phenotypic and functional heterogeneity are unique. DCs show a high expression of major histocompatibility complex class II molecules (MHC-II) and CD11c. They also express a lot of other molecules which allows their discrimination into different subtypes (59, 60).

Another unique characteristic of these immune cells is the ability of cross-presentation, a presentation of extracellular antigens in the context of major histocompatibility complex class I molecules (MHC-I) to activate naïve CD8^+^ T-cells for immunity against a lot of tumors and viruses that do not infect APCs (61, 62).

Through pathogens, cytokines and extracellular signals, such as pathogen-associated molecular patterns (PAMPs) and damage-associated molecular patterns (DAMPs), maturation of immature DCs is triggered (63, 64). Mature DCs secrete T-cell activating cytokines, increase MHC-II and CCR7 expression and decrease their endocytic activity (65–69). In addition to increased MHC-II expression, whilst the expression of other chemokine receptors is downregulated, DCs lose their adhesive structures during maturation, achieving the ability to migrate from the periphery to secondary lymphoid organs, where their antigens are presented to T-cells (70, 71).

Many T-cell immune checkpoint receptors have their ligands on APCs. Manipulation of DCs through checkpoint blockade hold great potential for avoiding T-cell anergy and inducing efficient antitumor immunity (72).

Programmed cell death 1 ligand 1 (PD-L1 also called B7-H1 or CD274) and PD-L2 (B7-DC or CD273) are expressed by DCs and other APCs. They inhibit cytokine production (IFN-γ, IL10) and proliferation of activated T-cells, which upregulate the inhibitory receptor programmed cell death 1 (PD-1) (73, 74). DCs with high expression of PD-L1 and PD-L2 can be found in the tumor microenvironment (TME) where engagement with the co-inhibitory receptor PD-1 limit the activity of effector T-cells (75–77). Blocking the interaction between PD-L1 and PD-1 as a tool in cancer immunotherapy has demonstrated therapeutic efficacy in several cancer types (78–80). Various studies showed remarkable anti-tumor effects in targeting PD-L1 in solid tumors with the engineered humanized antibody MPDL3280A (Atezolizumab), especially when PD-1 was expressed by tumor-infiltrating lymphocytes (TILs). However, the response rate has also been limited in several solid tumors (74, 78, 80).

CD80 is a member of the B7 superfamily and is expressed by DCs and T-cells too. On DCs it acts as a positive regulator after binding by CD28 and as a negative regulator when interacting with CTLA-4 on T-cells (81, 82). The checkpoint molecule CTLA-4 binds CD80, as well as CD86, with greater affinity and in a multivalent fashion compared to the co-stimulatory receptor CD28, which leads to the limitation of co-stimulatory signaling and thereby T-cell activation (83). Interestingly, PD-L1 of DCs additionally bind CD80 on T-cells and thereby inhibit T-cell responses (84). This means that there is a dual inhibitory effect of PD-L1 expression: first interaction between PD-L1 and PD-1 and second interaction between PD-L1 and CD80. Therapies with monoclonal antibodies against PD-1 in the treatment of cancer such as Nivolumab affect only the PD-L1/PD-1 pathway (79, 85). This alone may not lead to overcome anergy, but an anti-PD-L1 monoclonal antibody specific to the interaction between PD-L1 and CD80 seems to be able to prevent T-cell tolerance (86, 87). Further studies are required to determine whether monoclonal antibodies against PD-L1 or PD-1 are more effective. Expression of PD-L2 in tumor tissues and correlation to therapy failures targeting PD-1 are less well studied than PD-L1, but specific antibodies against PD-L2 could disrupt T-cell inhibition (88).

Inducible T-cell costimulatory-ligand (ICOS-L or CD275) expressed by DCs is a member of the B7 family of costimulatory ligands which has a sequence homology to CD80/CD86 and is important for T-cell regulation (89, 90). Blockade of ICOS-L disrupts binding to ICOS (CD278), which is an activating co-stimulatory checkpoint receptor up-regulated upon early T-cell activation (89, 91). ICOS is homologous to CD28 and CTLA-4, they all control T-cell activation and cytokine production (89, 91, 92). Interestingly, ICOS furthermore adjusts the immunological memory by CD40/CD40L dependent antibody class switching (93, 94). ICOS can be found in tumors of different cancer types like ovarian cancer and liver cancer, also expressed by TILs in CTLA-4 treated melanoma patients (95–97). The dual role, antitumor and protumor, could be a key for enhancement of antitumor immune responses by targeting the ICOS/ICOS-L pathway. There are several clinical trials with monoclonal antibodies against ICOS, for example with MEDI-570 (ClinicalTrials.gov: NCT02520791, NCT01127321) and JTX-2011 (Vopratelimab, NCT04319224, NCT02904226, NCT03989362, NCT04549025). Both promise potential in immune checkpoint inhibitory and antineoplastic activities by binding and blocking ICOS expressed on CD4^+^ TILs and thereby disrupt the binding on ICOS-L expressed by DCs. This prevents DC-induced proliferation and accumulation of regulatory ICOS^+^ T-cells and would also inhibit IL-10 production by CD4^+^ TILs.

For the development of anti-cancer therapies a greater understanding of DCs and their immune checkpoint ligands is needed. For example, combinations of DC vaccination and different immune checkpoint inhibitors hold great potential to activate naïve T-cells and induce immune memory responses in different cancer types on one hand and to activate effector T-cells in the TME on the other hand.

We have not yet included dendritic cells in our diagnostic panels.

## Immune Checkpoint Molecules

**Figure 5:**
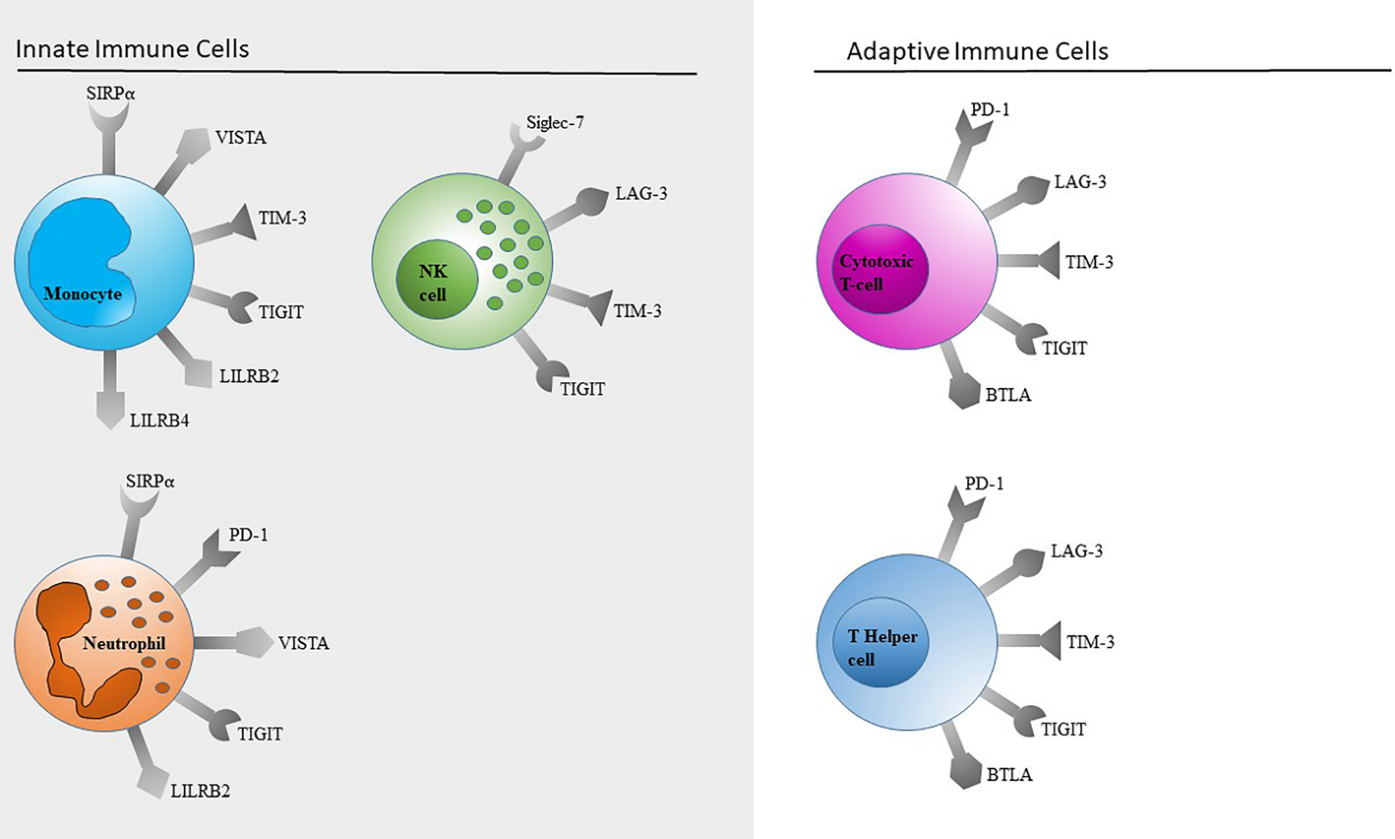
Schematic overview of immune checkpoints expressed on innate and adaptive immune cells. Only immune checkpoints included in our panels (**Table 1**) are shown. This selection is by no means a complete representation of all immune checkpoints.

For this review, we focused on checkpoint molecules for which we have established flow cytometric detection methods for several reasons (**Figure 5**). For most of our results, we were able to find further references in the literature. It was not possible for us to establish all the described detections, and we omitted PD-1 and CTLA-4 on T cells due to the broad data available.

### PD-1

An immune checkpoint that has already been integrated as a target in broad fields of clinical therapy is Programmed cell death 1 (PD-1). It is predominantly expressed on activated CD4^+^ and CD8^+^ T-cells. Moreover, it can be found on B-cells, NKT-cells, dendritic cells, and monocytes (98). There are conflicting reports on whether or not human NK cells express PD-1 (98, 99). So far, the FDA has approved three PD-1 inhibitors: Nivolumab, Pembrolizumab and Cemiplimab as well as three PD-L1 inhibitors: Atezolizumab, Avelumab, Durvalumab (100).

#### T-Cells

We too found that PD-1 is expressed on CD4^+^ and CD8^+^ T-cells of healthy adults with a percentage of about 33% and 31% respectively in unstimulated whole blood.

Interactions between PD-1 and its ligand PD-L1 keep cellular immunity from overreacting, maintain peripheral tolerance, and suppress the development of autoimmunity (101). However, T-cells that overexpress PD-1, exhibit low proliferation and cytokine production as well as low levels of cytokine release. They are described as so-called “exhausted” T-cells. Such an overexpression may result from permanent activation of the cellular immune system through chronic viral infection (102, 103). CD8^+^ tumor-infiltrating lymphocytes that express high levels of PD-1 have also been shown to be functionally impaired (104). Accordingly, both increased frequency of CD8^+^ PD-1^+^ T-cells and high PD-L1 expression levels can be looked at as negative prognostic factors in tumors like ovarian cancer (105).

#### Monocytes

PD-1 is expressed in low levels on monocytes (106) and can be upregulated upon toll-like receptor (TLR) stimulation (107, 108). As a negative immune checkpoint PD-1 inhibits activation of monocytes and thus reduces cytokine secretion, antigen presentation and phagocytosis. On one hand this mechanism prevents an overactivation of the immune system but on the other hand it leads to a reduced immune response in acute and chronic inflammatory conditions like sepsis, endocarditis, HIV (107, 109, 110) or cancer. We were not able to detect PD-1 on monocytes with our panel (**Table 1**).

### CTLA-4

Cytotoxic T lymphocyte antigen 4 (CTLA-4) (CD152) is an important member of the immunoglobulin-superfamily (111, 112). This family also includes CD28 and ICOS (stimulatory receptors) as well as PD-1, BTLA and TIGIT (inhibitory receptors). CTLA-4 downregulates the immune response after ligand binding. This inhibitory receptor and CD28 are homologous receptors expressed by CD4^+^ and CD8^+^ T-cells (113). Both share a pair of ligands: B7.1 (CD80) and B7.2 (CD86), which are expressed on the surface of antigen presenting cells (APCs) such as dendritic cells and B-cells (114). One dimer of CD28 can only bind one B7 dimer (one to one). One CTLA-4 dimer however, can bind two different B7 dimers, making the cross-linking bond much stronger than the single bond between CD28 and B7 molecules which leads to a much higher affinity and avidity (112, 115). This suggests that CTLA-4 preferentially interacts with B7 molecules and thereby aids in the limitation of immune response as a competitive inhibitor of CD28.

Binding of CTLA-4 to B7 molecules finally depends on their surface availability, which is a prerequisite for the receptors function as a negative regulator of proliferation and T-cells effector functions. Around 90% of CTLA-4 can be found in intracellular vesicles in FoxP3^+^ regulatory T-cells (T_reg_) or on the intracellular membrane of conventional T-cells. T-cell receptor signaling leads to activation, whereby CTLA-4 is rapidly expressed through exocytosis on the cell surface (81, 82). After binding of CTLA-4 to B7 it then interacts intracellularly with the tyrosine phosphatase SHP-2 and the serine/threonine phosphatase PP2A to inhibit T-cells (116, 117).

By using a flow cytometry assay Qureshi et al. observed a substantial transfer of CD86^+^ vesicles into CTLA-4^+^ cells. Their results indicate that CTLA-4 has a cell intrinsic function and seems to be able to capture and deplete its ligands by trans-endocytosis and thereby extrinsically inhibit T-cell activation *via* CD28 (118). Ipilimumab is the only FDA approved CTLA-4 inhibitor available to date (100).

### VISTA (VSIR, Gi24, Dies-1, PD-1H, B7-H5, C10orf54, SISP1, and DD1α)

V-domain Ig suppressor of T-cell activation (VISTA, also known as VSIR, Gi24, Dies-1, PD-1H, B7-H5, C10orf54, SISP1 and DD1α) was first described in 2011 as a new member of the Ig superfamily that has an inhibitory effect on T-cell activation (54).

VISTA is a type 1 transmembrane protein that consists of a single extracellular Ig-V domain, a stalk region, a transmembrane segment, and a cytoplasmic region without any signaling domains (ITAM, ITIM or ITSM motifs) (54). However, the cytoplasmic domain contains a Scr homology 2 (SH2)-binding motif, three C-terminal SH3-binding domains and multiple casein kinase 2 and phosphokinase C phosphorylation sites for signal transduction (119, 120). Structurally VISTA is associated with the B7-CD28 family and closest related to its members PD-L1 (regarding the Ig-V domain) or to PD-1 (regarding the cytoplasmic domain) (54, 121). Yet VISTA has several sequence features, which have not been identified in any other B7 family member, e.g., four additional invariant cysteines of which three are located within the Ig-V domain and one within the stalk region (54, 122).

VISTA is an important regulator of immune homeostasis and anti-tumor immunity. Within the immune cell compartment VISTA is mainly expressed by myeloid cells (neutrophils, monocytes, macrophages, and dendritic cells). Naïve T-cells and CD4^+^ T-cells express VISTA at lower levels, CD8^+^ T-cells, Foxp3^+^ T_reg_ and CD56^dim^ NK-cells show a minimal yet detectable expression, while CD56^bright^ NK-cells and B-cells are mostly VISTA negative (53, 54, 123).

#### T-Cells

VISTA functions as both, a receptor and a ligand depending on cellular context. Expressed by antigen presenting cells (APCs) and regulatory T-cells (T_reg_) VISTA as a ligand inhibits T-cell proliferation, cytokine and chemokine production, i.e., IFN-γ, IL-10, IL-17, IL-23 (54, 121, 124). The correspondent receptor on T-cells remains to be characterized. Expressed by conventional T-cells VISTA functions as a suppressive receptor. Antigen-specific T-cell responses are down-regulated through cell intrinsic signaling (121). Wang et al. identified V-set and Ig domain containing 3 (VSIG-3, IGSF11) as a potential ligand for VISTA (125). In addition to its inhibitory role, VISTA also has a co-stimulatory effect. Bharaj et al. described that in context of HIV, antigen-presentation by monocytes with high VISTA expression levels resulted in increased cytokine secretion by HIV-specific T-cells (126).

#### Monocytes

Lines et al. examined circulating blood cells by flow cytometry staining them with an anti-VISTA monoclonal antibody. They demonstrated that especially the myeloid compartment shows strong VISTA expression, and that VISTA appears to be expressed by all monocyte subsets: classical (CD14^++^CD16^-^), intermediate (CD14^+^CD16^+^) and non-classical (CD14^-^CD16^++^) (53). Several groups analyzed the impact of VISTA on innate immune cells in cancer, autoimmune and inflammatory diseases (54, 126–129).

### TIM-3

T cell immunoglobulin and mucin domain-containing protein 3 (TIM-3) is an inhibitory receptor and a transmembrane protein. It was originally described on T helpers cells type 1 (Th1) and cytotoxic T cells type 1 (Tc1) (130). TIM-3 has an extracellular IgV domain and a mucine stalk which consists of an N- and O-linked glycosylation site. The intracellular tail has tyrosine residues. The ligands galectin-9 and HMGB1 bind to TIM-3, which leads to a phosphorylation of two conserved tyrosine residues. The ligands Ceacam-1 and galectin-9 bind to different regions in the IgV domain but both ligands lead to the same phosphorylation of two tyrosine residues which are required for the functional activity of TIM-3 (131, 132). Another ligand, HLA-B-associated transcript 3 (Bat3), binds to the intracellular tail of TIM-3 and leads to a repression of TIM-3’s function. Bat-3 prevents TIM-3 dependent cell death and exhaustion. It saves Th1 cells from galectin-9 mediated cell death and stimulates proliferation and pro-inflammatory cytokine production (132). TIM-3 is part of the TIM gene family as well as Tim-1 and Tim-4. Besides T-cells it is expressed on NK-cells, monocytes, macrophages and DCs (133).

#### T-Cells

**Figure 6:**
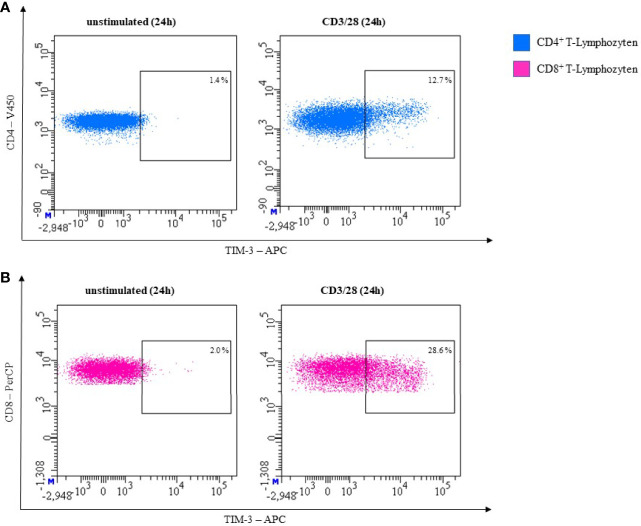
Representative flow cytometric analysis of TIM-3 expression on T helper cells (CD4^+^) **(A)** and cytotoxic T cells (CD8^+^) **(B)**. Comparison of unstimulated (left) and CD3/28 stimulated results after 24h (right) (healthy donor, male, 23 years old).

In our own laboratory we observed very low expression levels of TIM-3 on both unstimulated CD3^+^CD4^+^ and CD3^+^CD8^+^ T-cells. After stimulating the T-cells with CD3/28 for 24 hours the expression of TIM-3 was upregulated. This is shown in **Figure 6** for CD3/28 stimulated T cells.

#### NK-Cells

**Figure 7:**
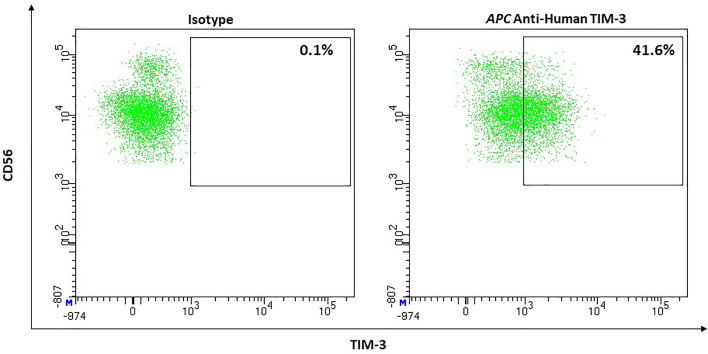
Representative flow cytometric analysis of the expression of the immune checkpoint TIM-3 on resting NK cells of a healthy donor (male, 23 years old) compared with isotype control.

NK-cells are the lymphocyte population with the highest surface expression of TIM-3. CD56^dim^ NK-cells express the checkpoint with higher frequency than CD56^bright^ NK-cells (72% ± 5% *vs.* 53% ± 6% [P <.001, n = 20]) and TIM-3’s surface expression also appears to be slightly denser on the mature subset (134) (**Figure 7**). Stimulation with IL-2, IL-12, IL-15, and IL-18 results in an up-regulation of TIM-3 (134, 135). TNF-α was also reported to increase surface expression through an NF-κB signaling pathway (136). Eomes and T-bet, two transcription factors, play an important role in regulating TIM-3 on T-cells. In NK-cells regulation through T-bet appears to be more important (134, 137). While TIM-3 was described as a marker of exhaustion in the context of advanced melanoma (138) and other advanced tumors (139), TIM-3^+^ NK-cells from healthy donors do show functional diversity thus suggesting that TIM-3 cannot be looked at as an independent exhaustion marker in NK-cells (140). There have been conflicting reports on TIM-3’s function in the context of NK-cells. Gleason et al. reported that engagement of TIM-3 increased IFN-γ production (134). They proposed activation of ERK followed by degradation of IκBα as the responsible signaling pathway. Others reported TIM-3 to be an inhibitory receptor capable of restricting NK-cells potential to lyse target cells and to produce IFN-γ (135, 138). Gleason et al. discussed the possibility that the receptor could very well function both as activator and as inhibitor. This could be realized through phosphorylation of different tyrosine residues in the cytoplasmic tail, which then could lead to distinct adaptor proteins being recruited, ultimately resulting in different pathways. They named the surrounding microenvironment and ligand-dependence (as is the case with Tim-1) as possible factors that can decide which distinct receptor function is triggered (134). In contrast to T-cells, chronic activation of TIM-3 does not result in apoptosis (138).

#### Monocytes

TIM-3 is constitutively expressed on unstimulated peripheral blood CD14^+^ monocytes. Zhang et al. (108) used flow cytometry to analyze PBMCs from healthy humans for TIM-3 surface expression on naïve and stimulated monocytes. They further examined intracellular expression of IL-12, -10, -6, and TNF-α, proinflammatory cytokines produced by monocytes. They showed that unstimulated monocytes with low or nearly no cytokine expression, express TIM-3 at relatively high levels. This indicates TIM-3’s inhibitory role in monocytes. During the first 24h after stimulation with 5µg/ml LPS they observed a rapid reduction of TIM-3’s expression, that resolved slowly after 48h. Additionally the LPS mediated decline in TIM-3 expression correlated inversely with IL-12 release. To verify that this effect is due to TIM-3 expression on monocytes, its expression was blocked with a monoclonal antibody confirming the increase of TLR-mediated IL-12 production in monocytes. Thus, downregulation of TIM-3 might play an important role in inflammatory conditions.

Other studies show similar results for TIM-3 expression under TLR Stimulation. Ma et al. (141) stimulated monocytes with 1 µg/ml LPS for 1-6 h. TIM-3’s surface expression was at first reduced and almost not existing after 6 h of stimulation.

Anderson et al. generated an antagonistic antibody of TIM-3 showing a rapid reduction in galactin-9 mediated TNF-α production in monocytes suggesting that TIM-3 could promote production of pro-inflammatory cytokines such as TNF-α in monocytes (142). Therefore, it may be an important therapeutic target in inflammatory diseases. Interestingly, these results are in contradiction with the results of Zhang et al. (108). Further studies are needed to evaluate influence of TIM-3 on cytokine production in monocytes.

#### Neutrophils

To our knowledge, no studies have been performed on TIM-3 expression on neutrophils. We could not detect any relevant TIM-3 expression on neutrophils in unstimulated whole blood (**Figure 4**).

### LAG-3

The first description of Lymphocyte-activation gene 3 was in 1990 on activated NK- and T-cells (143). Furthermore, LAG-3 can be detected on B-cells (144) and dendritic cells (145). LAG-3 contains 4 extracellular domains. There are strong internal homologies between domain 1 and 3, as well as domain 2 and 4. The peptide sequence and the general organization of the molecule lead to the assumption that LAG-3 is closely related to CD4. Furthermore, they both share a location in the distal part of chromosome 12 (143). The cytoplasmic tail of LAG-3 has a unique KIEELE motif (131). There is a correlation between the expression level and the inhibitory function of LAG-3. An FXXL motif in the membrane-proximal region and a C-terminal EX repeat transduce two inhibitory signals of LAG-3 which inhibit IL-2 production. They are independent from each other. LAG-3 could be another target for combinatorial therapy because other inhibitory co-receptors do not use these motifs (146). Major histocompatibility complex class II (MHC-II) is the main ligand of LAG-3. Fibrinogen-like protein (FGL1) is a liver secreted protein which inhibits antigen-specific T-cell activation. It is another functional ligand of LAG-3 and works independently from MHC-II. The removal of FGL1 promotes T-cell immunity (147). LSECtin, a Type-II transmembrane protein of the C-type lectin-superfamily is also able to interact with LAG-3 and thus cause inhibition of INF-γ production by effector T-cells. LSECtin is expressed in the liver but can also be found in tumor tissues like melanoma (148).

#### T-Cells

**Figure 8:**
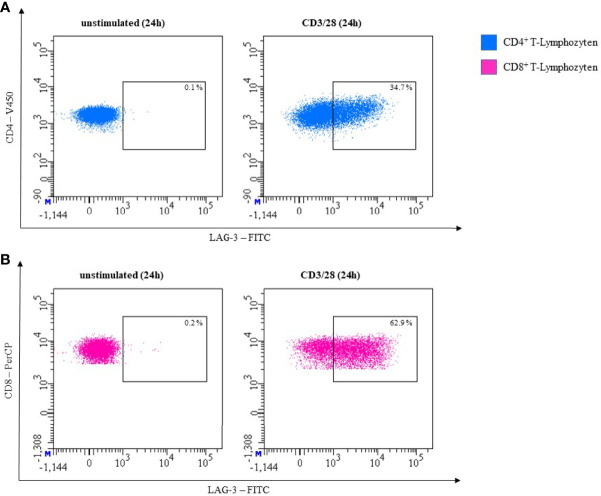
Representative flow cytometric analysis of LAG-3 expression on T helper cells (CD4^+^) **(A)** and cytotoxic T cells (CD8^+^) **(B)**. Comparison of unstimulated (left) and CD3/28 stimulated results after 24h (right) (healthy donor, male, 23 years old).

In our own experiments, we did not observe LAG-3 expression on unstimulated CD3^+^CD4^+^ or CD3^+^CD8^+^ T-cells. Expression on both subsets increased after 24h of stimulation with CD3/28. In **Figure 8**, effect of CD3/28 stimulation of T-cells is shown.

#### NK-Cells

**Figure 9:**
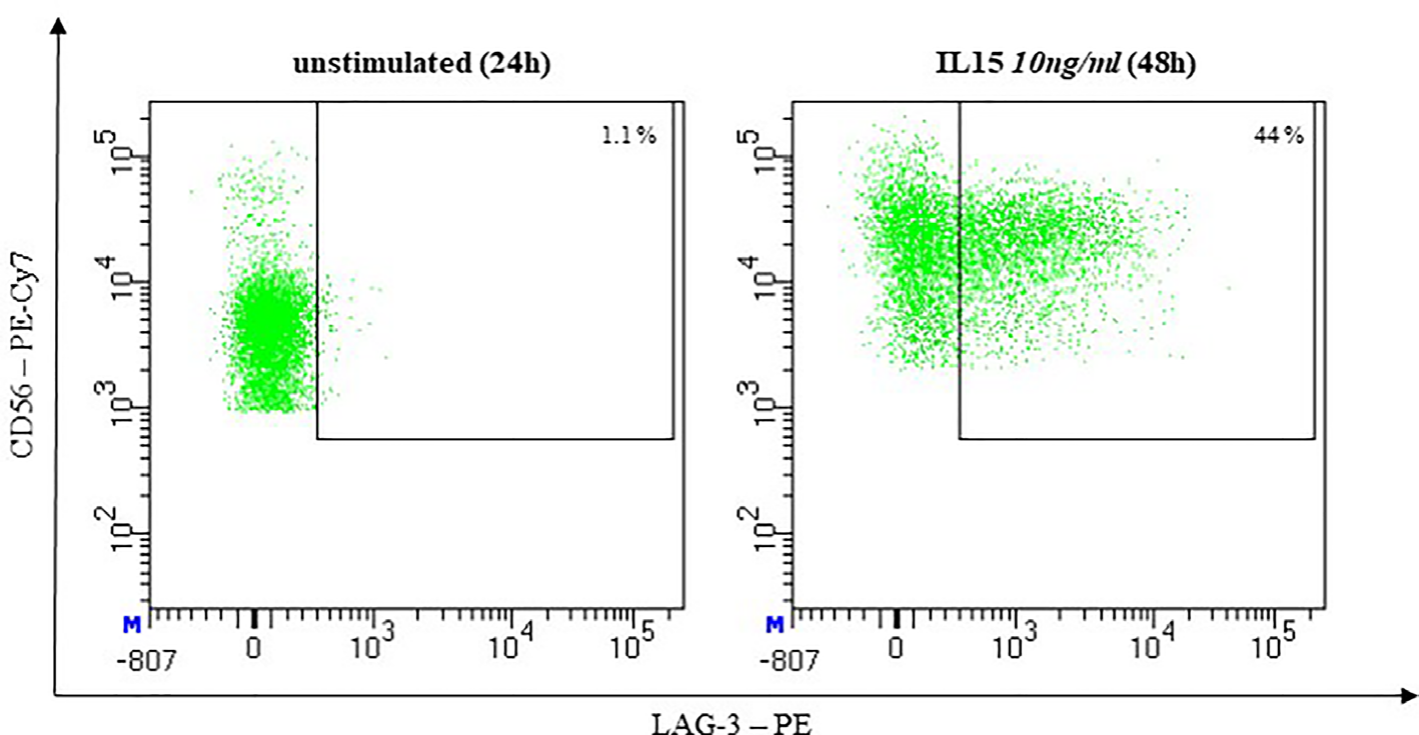
Representative flow cytometric analysis of LAG-3 Expression on NK cells. Comparison of unstimulated NK cells after 48h of co-incubation with complete medium (left) and stimulated NK cells after 48h of co-incubation with 10ng/ml IL-15 (right). (healthy donor, female, 65 years old).

Lymphocyte activation gen (LAG)-3 was described as undetectable on resting but expressed on activated NK-cells (143) (**Figure 9**).

Our understanding of LAG-3’s functional role on NK-cells is still developing. NK-cells from homozygote LAG-3^-/-^ mice show reduced cytotoxic activity against different tumor cell lines but remain able to lyse MHC class-I deficient targets (149). However, when Huard et al. used two different monoclonal antibodies (mAbs) or a soluble form of LAG-3 to inhibit interaction between LAG-3 and its ligand MHC class II, they did not observe any changes in their cytotoxic activity against different targets. They therefore concluded that LAG-3 is not involved in the regulation of NK-cell cytotoxicity. However, they did not investigate whether LAG-3 could impact cytokine secretion in any form (150).

### TIGIT (VSig9, Vstm3, WUCAM)

TIGIT, which stands for “T-cell Ig and ITIM domain”, was first described in 2009 (151–153). The member of the Ig superfamily consists of a single extracellular immunoglobulin domain, a type 1 transmembrane region and a cytoplasmatic tail with a single immunoreceptor tyrosine based inhibitory motif (ITIM) and an immunoglobulin tail tyrosine (ITT)-like motif. It is expressed by activated T-cells, T_reg_, memory T-cells, and NK-cells (153).

All known TIGIT ligands are Nectins and Nectin-like molecules (Necls), which are cell adhesion molecules. CD155 (a.k.a. Poliovirus receptor [PVR], Necl-5) shows the highest affinity, while CD112 (a.k.a. PVRL2, Nectin-2) only binds with low affinity. Yu et al. also reported CD113 (a.k.a. PVRL3) to be a TIGIT ligand which Stanietsky et al. were not able to confirm (152, 153). Recently Nectin4 has been identified as an additional TIGIT-ligand (154). CD155 is expressed on T, B, NK and NKT-cells, DCs, macrophages, granulocytes, and monocytes as well as on non-hematopoietic cells like endothelia and epithelia cells or on cells of the central nervous system (155). Furthermore, CD155 can be overexpressed in human malignancies like primary lung adenocarcinoma (156), pancreatic cancer (157), primary melanoma and metastasis of melanoma (158). In all those cases overexpression correlates with poor prognostic factors. Patients with different types of cancer also show increased levels of soluble CD155 in their serum (159). CD112 is expressed on macrophages, DCs, granulocytes and monocytes (155) but also on malignant cells like acute myeloid leukemia (AML) blasts (160). Nectin4 expression in various healthy tissues ranges from weak to moderate but can be highly expressed in tumors like bladder-, breast- or pancreatic cancer (161). In patients with gastric cancer, overexpression of Nectin4 was associated with poor prognostic factors like, low differentiation, primary tumor size, lymph node metastasis and higher TNM staging as well as shorter overall survival (162).

Both CD155 and CD112 are also recognized by the activating Receptor CD226 [a.k.a. DNAXaccessory molecule-1 (DNAM-1)] (163). CD96 (a.k.a. T-cell activated increased late expression [Tactile]) also binds CD155, but its functional role in humans is not well characterized (164). Due to its higher affinity, TIGIT (Kd = 1-3 nM) can block interaction between CD155 and CD266 or CD155 and CD96 (153). To add even more complexity to this regulatory network, CD112R [a.k.a. poliovirus receptor related immunoglobulin domain containing (PVRIG)] is another inhibitory receptor, that also binds CD112 as its ligand (165). Nectin4 interacts with TIGIT but not with CD266, CD96 or CD112R (154).

#### T-Cells

In healthy individuals, about 13% of CD4^+^ and 24% of CD8^+^ T-cells express TIGIT in unstimulated whole blood samples.

TIGIT competes with CD226 for the common ligand CD155. The higher affinity favors the inhibitory counterpart, which results in reduced T-cell proliferation and cytokine production. This is transmitted through a reduced expression of T-bet (T-box expressed in T-cells), IRF4 (Interferon regulatory factor 4), and RORc (retinoic acid receptor [RAR] related orphan receptor gamma) (166).

TIGIT is upregulated on dysfunctional CD8^+^ cells that can especially be found in the tumor microenvironment. For example, CD8^+^ TIGIT^+^ T-cells were found in patients with multiple myeloma. The ability of those cells to proliferate and degranulate inflammatory cytokines was shown to be insufficient (167).

**Figure 10:**
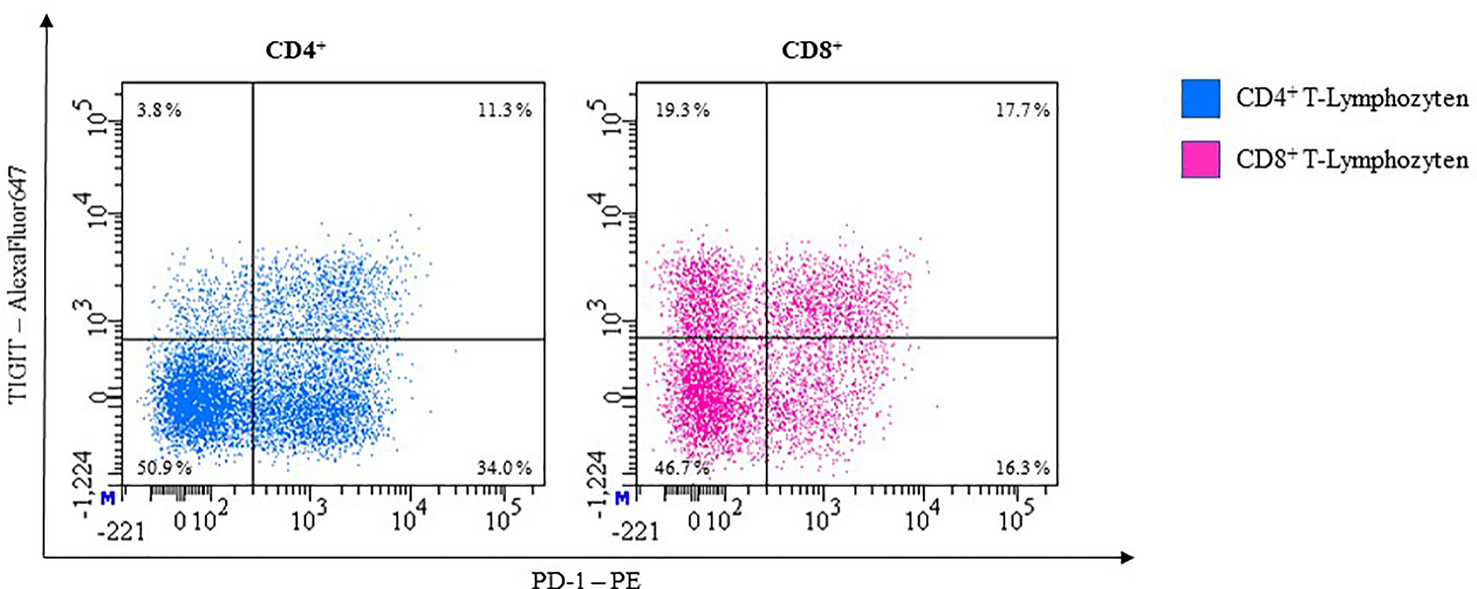
Representative flow cytometric analysis of the expression of immune checkpoints TIGIT and PD-1 on unstimulated whole blood T lymphocytes of a healthy 41-year-old female.

Dual blocking TIGIT and PD-1 can partly restore the capacities of CD8^+^ T-cells (168, 169). Further studies that aim at establishing an anti-TIGIT monoclonal antibody (mAb), are at different stages of testing. With PD-1 and TIGIT both being expressed on the T-cell surface (170), measurement is possible through cell surface staining with antibodies in flow cytometry (**Figure 10**).

#### NK-Cells

Stanietsky and colleagues were the first group to establish TIGITs role as an inhibitory receptor on natural killer (NK) cells (152).

**Figure 11:**
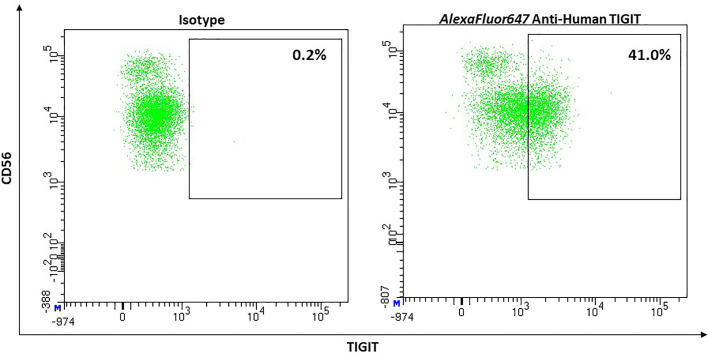
Representative flow cytometric analysis of the expression of the immune checkpoint TIGIT on resting NK cells of a healthy donor (male, 23 years old) compared with isotype control.

Its expression on NK-cells shows a big interindividual variance, ranging from 20% to up to 90% (mean, 62.57%), with TIGIT expression being higher on CD56^dim^ than CD 56^bright^ NK-cells (171) and **Figure 11**.

Interaction between PVR and TIGIT results in phosphorylation of Tyr225 in the ITT-like motif by Src family kinases Fyn or Lck initiating two known signaling pathways:

i)cytosolic adaptor protein Grb2 binds to phosphorylated TIGIT (pTIGIT) and recruits SH2-containing inositol phosphatase 1 (SHIP1). SHIP1 then inhibits PI3K by hydrolysis of PI(3,4,5)P3, inactivating its downstream effectors including parts of the mitogen−activated protein kinase (MAPK) pathway, ultimately resulting in a disruption of the polarization of granules toward the immunological synapse between NK and target cells, almost blocking NK-cell-mediated cytolysis (172).
ii) adaptor protein β-arrestin 2 binds to pTIGIT and recruits SHIP1. SHIP1 suppresses auto-ubiquitination of TRAF6 which then impairs activation of NF-κB. In consequence, secretion of IFN-γ by NK-cells is inhibited (173).

Based on research with mice, He et al. proposed that TIGIT could also play a role in the process of NK-cell education, that is separate from the MHC-I dependent education pathway and that also does not relay on involvement of CD226 (155).

#### Monocytes

TIGIT expression on monocytes is controversial and unclear. There are studies negating the expression on resting and activated monocytes (153). However, studies by Luo et al. describe TIGIT expression on a small percentage of monocytes in healthy individuals and showed that there might be a tendency for a higher percentage of TIGIT expressing monocytes in autoimmune diseases such as rheumatoid arthritis and systemic lupus erythematosus (174, 175).

In our experiments we detected low TIGIT expression on monocytes compared to isotype control in healthy individuals (**Figure 3**). Further studies are needed to create a consistent picture of the TIGIT expression on monocytes.

#### Neutrophils

To our knowledge, no studies have been performed on TIGIT expression on neutrophils. We show that TIGIT is expressed at a low level on neutrophils in unstimulated whole blood (**Figure 4**).

### SIRPα (CD172a, PTPNS1, MFR, p84, BIT, SHPS-1)

Signal regulatory protein alpha (SIRPα) was first described in 1996 as a novel membrane-associated glycoprotein and potential substrate for Src homology 2 (SH2)-containing protein tyrosine phosphatases, SHP-1 and SHP-2 in rat fibroblasts (176).

SIRPα contains three Ig like domains – one N-terminal V-set domain and two C1-set domains, a transmembrane segment and a cytoplasmic region with two ITIM motifs containing four tyrosine residues (176–178).

SIRPs form an own family of paired receptors. SIRPα, β1 and γ share structurally closely related extracellular regions but show diversity within their transmembrane and cytoplasmic regions and thus facilitate different intracellular signals. SIRPα has an inhibitory effect, SIRPβ1 has an activating effect and SIRPγ has no signaling function [reviewed in (179)].

CD47 (also known as Integrin-associated protein, IAP) was identified as a ligand for SIRPα (180). CD47 and SIPRα however are not restricted to interact with each other but are both known to have alternative binding partners. SIRPα is involved in inhibiting alveolar macrophage phagocytosis through interaction with lung surfactant proteins SP-A and SP-D (181) while CD47 interacts with several integrins and functions as a receptor for thrombospondin-1 (182, 183). This review focuses on the SIRPα-CD47 axis.

As CD47 is ubiquitously expressed including erythrocytes and thrombocytes, it was initially characterized as a ‘marker of self’ (184). Also, senescent erythrocytes have shown to undergo CD47 conformational changes leading to engulfment by splenic macrophages (185). Consequently, CD47-SIRPα interaction was classified as a ‘do not eat me’ signal preventing inadequate phagocytosis.

The interaction between SIRPα on macrophages and CD47 leads to phosphorylation of SIRPα’s ITIM motifs involving recruitment of SHP-1 and SHP-2. Subsequently, accumulation of non-muscle myosin IIA at the phagocytic synapse is inhibited compromising contractile engulfment (186).

Within the immune cell compartment SIRPα is highly expressed by myeloid cells (macrophages, monocytes, granulocytes, dendritic cells) while T-cells, B-cells and NK-cells do not show any relevant SIRPα expression (55).

#### Monocytes

Adams et al. analyzed the SIRPα expression on rat monocytes finding high surface expression levels (177).. Seiffert et al. showed similar results in a study on cells from healthy human donors. They incubated monocytes with agonistic anti-SIRPα monoclonal antibodies and observed the expression using flow cytometry. Compared to other hematological cells, monocytes had the strongest SIRPα expression (55). Smith et al. confirmed the constitutive SIRPα expression on monocytes using flow cytometry as well (187).

### BTLA

B and T lymphocyte attenuator (BTLA) is an inhibitory receptor expressed by B- and T-cells (188). It is a cell surface molecule (189). BTLA is an immunoglobulin domain containing glycoprotein and has two immune receptor tyrosine based inhibitory motifs (190).

It has been indicated that BTLA is recognized by B7x which is an orphan B7 homolog (191). Other studies reported herpesvirus entry mediator (HVEM) as another ligand for BTLA. The extracellular immunoglobulin domain of BTLA is connected with the membrane distal cysteine-rich domain (CRD1) of herpesvirus entry mediator (HVEM) (192). HVEM is part of the TNFR superfamily, a type 1 membrane protein with a N terminal extracellular region. The cytoplasmic segment is closely associated with TNFR- associated factors (TRAFs) and in addition with STAT3 signaling pathways (193, 194).

#### T-Cells

There is no expression of BTLA on naive T-cells. The expression of BTLA is induced in activated T-cells and remains on T-helper type 1 Th1 but not on Th2 cells. Activation of BTLA leads to phosphorylation of its tyrosine and linkage to Src homology domain 2 (SH2). Furthermore, it lessens the CD3 induced Interleukin 2 (IL-2) production. BTLA reduces the proliferation of T-cells (190).

Complementarily to its inhibitory function, other studies show an activating feature. BTLA on CD8^+^ dendritic cells acts as a trans-activating ligand and delivers positive co-signals through HVEM expression in T-cells. HVEM-BTLA interaction triggers a bidirectional co-signaling system in virus defense by amplifying the differentiation of memory CD8^+^ T-cells (195).

### Siglec-7

Sialic acid-binding immunoglobulin-like lectin 7 (Siglec-7, a.k.a. p75/AIRMI, CD328) was first identified in 1999 by Falco et al. (196). They called this 75-kD glycoprotein p75/AIRM1 (adhesion inhibitory receptor molecule 1). In the same year, Nicoll et al. correctly categorized it as a member of the Siglec family (56).

This family of surface transmembrane receptors belongs to the immunoglobulin superfamily and consists of 14 members that have been identified in humans. They can be further divided into one group of Siglecs that are conserved across mammals and a second group, the CD33-related Siglecs, whose members vary among mammals. Siglec-7 belongs to the latter.

All Siglecs bind sialylated glycans but each with a distinct preference. Sialylated glycans can be found on all mammalian cells and are thus regarded as markers of self. They form in the golgi apparatus where different sialyltransferases transfer sialic acids to the terminal ends of glycoproteins and glycolipids. Siglecs can either interact with sialylated glycans on other cells (trans) or with sialylated glycans on the same cell (cis). Most of the Siglecs contain an ITIM-motif in their cytoplasmic tail and thus provide inhibitory signaling. However, Siglec-14, -15 and -16 associate with the DAP12 adaptor which contains an ITAM, hence they provide an activating signal (197, 198).

Siglec-7 is a type 1 membrane protein. Its extracellular region consists of three Ig-like domains: one N-terminal V-set domain and two C2-set domains. A transmembrane region links the extracellular region to the cytoplasmic tail that includes a membrane proximal ITIM- and a membrane-distal ITIM-like motif (56, 196). Siglec-7 binds terminal α2,3 and α2,6-linked sialic acids with moderate affinity but shows preferred binding to α2,8-disialic acid and branched α2,6-sialylated glycans (199). Interaction with its ligands results in a polarization of Siglec-7 towards the immunologic synapsis and increased phosphorylation of the ITIM motif, which than allows the recruitment of SHP-1. Ultimately, the interaction reduces both chemokine production and cytolytic potential towards the target cell (200). However, interactions between the membrane proximal ITIM motif and SHP-1 and -2 are not just essential to forward the inhibitory signal but could also influence ligand recognition by Siglec-7 in an “inside out” signaling fashion. This possibility was raised because mutations in the ITIM-motif can cause increased binding between Siglec-7 and its ligands (201).

Disialosyl globopentaosylceramide (DSGb5) is an internally branched α2,6-linked disialic ganglioside that is expressed on renal carcinoma cells (RCC) and its expression correlates with higher rates of distant metastasis. Interaction between DSGb5 and Siglec-7 reduced cytotoxicity of NK-cells towards RCC cells *in vitro* (202).

GD3 is a ganglioside with α2,8-disialic acid overexpressed on melanoma cells and is also able to inhibit NK-cell cytotoxicity through interaction with Siglec-7 (203).

Both ligands were not capable to interact with Siglec-7 if it was masked by cis-interaction with endogenous ligands. Pretreatment of the NK-cells with neuraminidase was required to unmask the receptor, which enabled the receptor to interact with its ligand and ultimately inhibit the NK-cell mediated killing of targets. Jandus et al. also observed a consistent expression of Siglec-7 ligands in AML and chronic lymphocytic leukemia patients as well as in melanoma patients, where the expression was restricted to malignant cells only (204). However, they reported that ligand expression on malignant cells was able to inhibit the antitumor response by NK-cells directly without sialidase pretreatment. Siglec-7 is expressed by NK cells, monocytes, macrophages, and neutrophils (197).

#### NK-Cells

**Figure 12:**
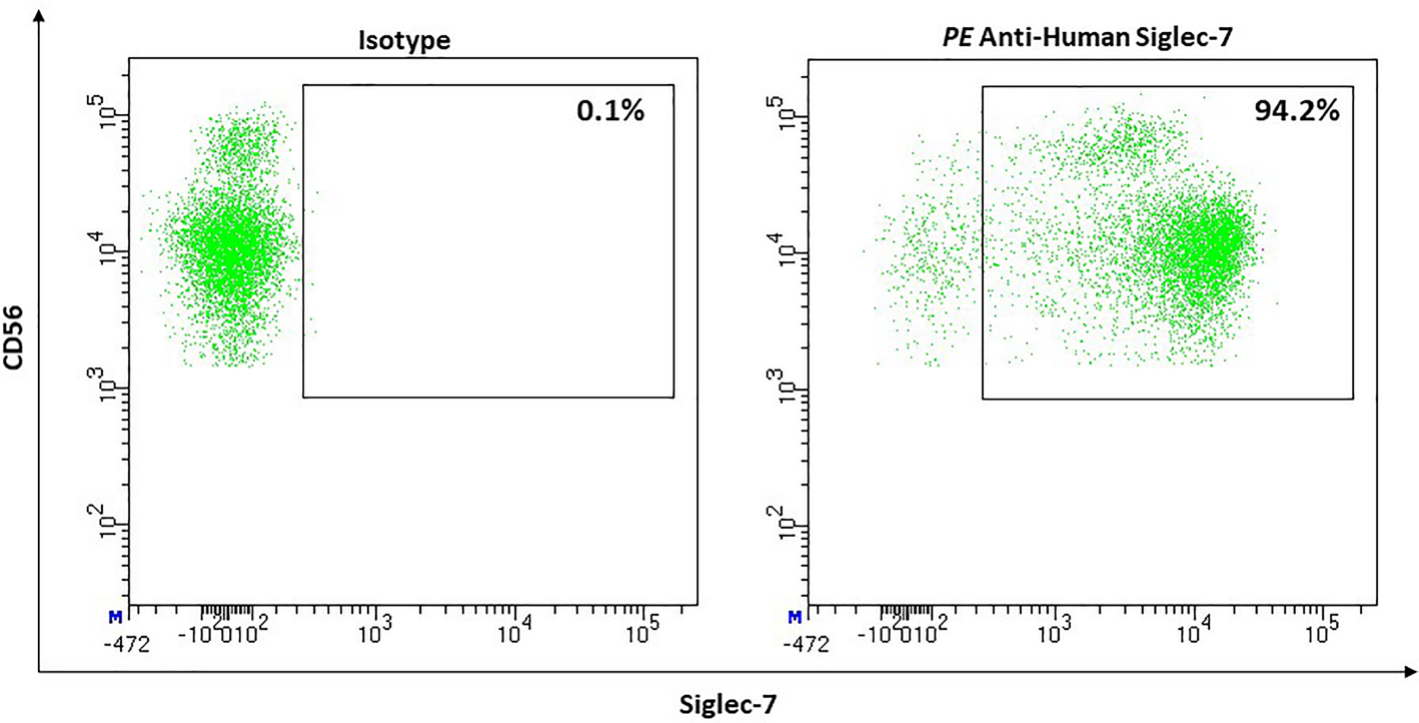
Representative flow cytometric analysis of the expression of the immune checkpoint Siglec-7 on resting NK cells of a healthy donor (male, 23 years old) compared with isotype control.

Most NK-cells express Siglec-7 in healthy humans (median, 80.6%; 95% CI, 70.57–90.63) (**Figure 12**). Expression on mature CD56^dim^ NK cells appears to be more dens than on CD56^bright^ NK cells (205). However, CD56^bright^ NK-cells show a higher density of sialic acids on their cell surface compared to CD56^dim^. This led to the suggestion that masking effects could be stronger on CD56^bright^ than on CD56^dim^ NK-cells (206). Although Siglec-7 is an inhibitory receptor, the absence of Siglec-7 defines a more dysfunctional subset of NK-cells. Siglec-7^+^ cells express activating receptors (e.g., CD16, CD38, DNAM1, NCRs) more frequently and show a higher ability to degranulate and to produce IFN-γ than Siglec-7^-^ NK-cells (205).

Interestingly, obesity as a risk factor for infections and several cancer types, influences the Siglec-7 expression on NK-cells: the CD56^bright^ subset shows a reduction in Siglec-7 surface density. Nevertheless, the overall frequency of Siglec-7^+^ NK-cells in the peripheral blood remains normal (206).

### LILRB2 (ILT 4, CD85d)

A further family of immune checkpoint receptors are the leukocyte Ig-like receptors (LILR), also known as Ig-like transcript (ILT) or CD85. They belong to the immunoglobulin superfamily (IgSF) and can be divided into immune system activating (207) and inhibitory receptors (208).

In this review, we will focus on two inhibitory members of the LILR family: Leukocyte immunoglobulin-like receptor superfamily B (LILRB) 2 and LILRB 4. They are type 1 transmembrane glycoproteins, that consist of extracellular immunoglobulin-like domains responsible for ligand binding, a transmembrane domain and a cytoplasmatic tail with immunoreceptor-tyrosine based inhibitory motifs (ITIM). The tyrosines contained in the ITIMs are phosphorylated by kinases, e.g., Src-kinase. Subsequently, phosphatases like SHP-1, SHP-2 or SHIP can bind to these phosphotyrosines with their SH2-domains. This interaction results in phosphatase activation. The activated phosphatases are able to dephosphorylate intracellular molecules that activate different intracellular signaling cascades leading to downregulation of the immune response. This explains how LILRB2 and LILRB4 function as negative immune checkpoints and mediate inhibition of immune cell activation (209, 210).

Using flow cytometry, Fanger et al. analyzed the expression of LILRB2 on circulating blood lymphocytes, monocytes and dendritic cells showing that LILRB2 cannot be found on B-cells, T-cells and NK-cells but is highly expressed on monocytes and dendritic cells (211).

LILRB2 binds to classical and non-classical HLA class I (212), members of the angiopoietin-like protein family (213), and β-Amyloid oligomers (209).

#### Monocytes

Venet et al. confirmed that circulating monocytes from healthy donors express LILRB2 at high levels. Furthermore, they described that CD16^+^ monocytes show a significantly higher LILRB2 expression than CD16^-^ monocytes, indicating that especially nonclassical proinflammatory CD16^+^ monocyte may play a role in dysregulating immune responses and altering the monocyte phenotype in inflammatory conditions (214).

#### Neutrophils

Baudhuin et al. were the first to elaborately analyze LILRB2 on neutrophils. The preferred ligand for LILRB2, HLA-G, has two other well-known receptors namely LILRB1 (ILT2, CD85j) and KIR2DL4 (215). The authors described that neither LILRB1 nor KIR2DL4 were expressed by neutrophils, leaving LILRB2 as the only known receptor for HLA-G expressed by neutrophils. On resting neutrophils, they detected high LILRB2 surface expression (68,8 ± 19,1%) and localized a pool of LILRB2 within neutrophil granules. LILRB2 stored in those intracellular granules was mobilized to the surface through exocytosis upon stimulation with fMLF, LPS or TNF-α resulting in increased surface expression. Up-regulation occurred rapidly reaching a plateau after 15 min. Furthermore, in a model with the myelomonoblast PLB-985 cell line, Baudhuin et al. identified LILRB2 expression as a process induced during neutrophil differentiation.

Functionally, LILRB2-HLA-G interaction has shown to inhibit neutrophil phagocytic function and CD32a-mediated production of reactive oxygen species. The corresponding signaling pathway in neutrophils has not been analyzed, but regarding studies performed with monocytes, Baudhuin et al. suggested that LILRB2-HLA-G interaction might induce SHP-1-mediated deactivation of the spleen tyrosine kinase (Syk). Syk is important for calcium mobilization and neutrophil activation. Finally, Baudhuin et al. performed an *in vitro* experiment incubating healthy neutrophils with either healthy or septic plasma. LILRB2 up-regulation upon stimulation was dysregulated under sepsis conditions (215).

Venet et al. performed a study evaluating LILRB2 expression by monocytes and neutrophils in septic shock patients. In comparison to healthy controls, LILRB2 expression on neutrophils was significantly increased in septic shock patients (214).

### LILRB4 (ILT 3, CD85k)

We have already briefly introduced this receptor in 3.10.; Cella et al. analyzed its expression on hematological cells by monoclonal antibody staining. B-cells, T-cells and NK-cells could not be stained in contrast to monocytes, dendritic cells, monocyte-derived dendritic cells, and macrophages (216).

#### Monocytes

CD14^+^ monocytes and THP-1, a myelo-monocytic cell line from an AML patient, express LILRB4 on the cells’ surface (216). Other studies found that monocytes circulating in cerebral spinal fluid express LILRB4 at higher levels than peripheral blood monocytes (217). Further, Cella et al. confirmed the role of LILRB4 expressed on monocytes as a negative immune regulator (216). They triggered monocytes with anti-HLA-DR or anti-FcγRIII, which would normally induce intracellular Ca^2+^ release. Yet when they stimulated LILRB4 in parallel, this could be inhibited. The ligand of LILRB 4 is unknown (209).

Lu et al. also demonstrated LILRB4s inhibitory function. They incubated THP-1 cells with the monocyte activator CD64 (anti-CD64) alone or co-ligated with LILRB4 (anti-LILRB4). LILRB4 co-ligation resulted in a significant decrease in CD64-induced production of pro-inflammatory TNF-α. The underlying mechanism described is the LILRB4 induced inhibition of CD64-mediated phosphorylation of signal molecules important in cell activation cascades. These results thus assume that CD64-mediated activation of monocytes can be inhibited by LILRB4 (218).

Kim-Schulze et al. found that membrane-bound and soluble LILRB4 inhibits T-cell proliferation, can anergize CD4^+^ T cells, and is able to suppress differentiation of CD8^+^ cytotoxic T-cells. On the other hand, LILRB4 promotes differentiation of immune system restraining CD8^+^ suppressor T-cells which upregulates LILRB4 on monocytes and dendritic cells making them tolerogenic (219). Another study by Chang et al. showed similar results (220). They showed that CD8^+^ CD28^-^ T-suppressor cells induce upregulation of both LILRB2 and LILRB4 on antigen presenting cells (APC) such as monocytes and dendritic cells. Therefore, they incubated monocytes and immature dendritic cells with T-suppressor cells from generated T-cell lines. On APC pretreated with T-Suppressor cells, surface expression of LILRB2 and 4 was upregulated while the co-stimulatory CD86 was downregulated. T-suppressor cells upregulated inhibitory receptors on APC (220). Further they generated myelomonocytic cell lines (KG1) overexpressing LILRB2 and LILRB4 and could show that this overexpression reduces CD4^+^ T-cell mediated upregulation of co-stimulatory receptor CD80. These results support their hypothesis that LILRB2 and 4 lead to T-cell anergy and induce immune tolerance. *In vivo* experiments with blood from patients after heart transplantation present similar results (220).

## Pathology

**Figure 13:**
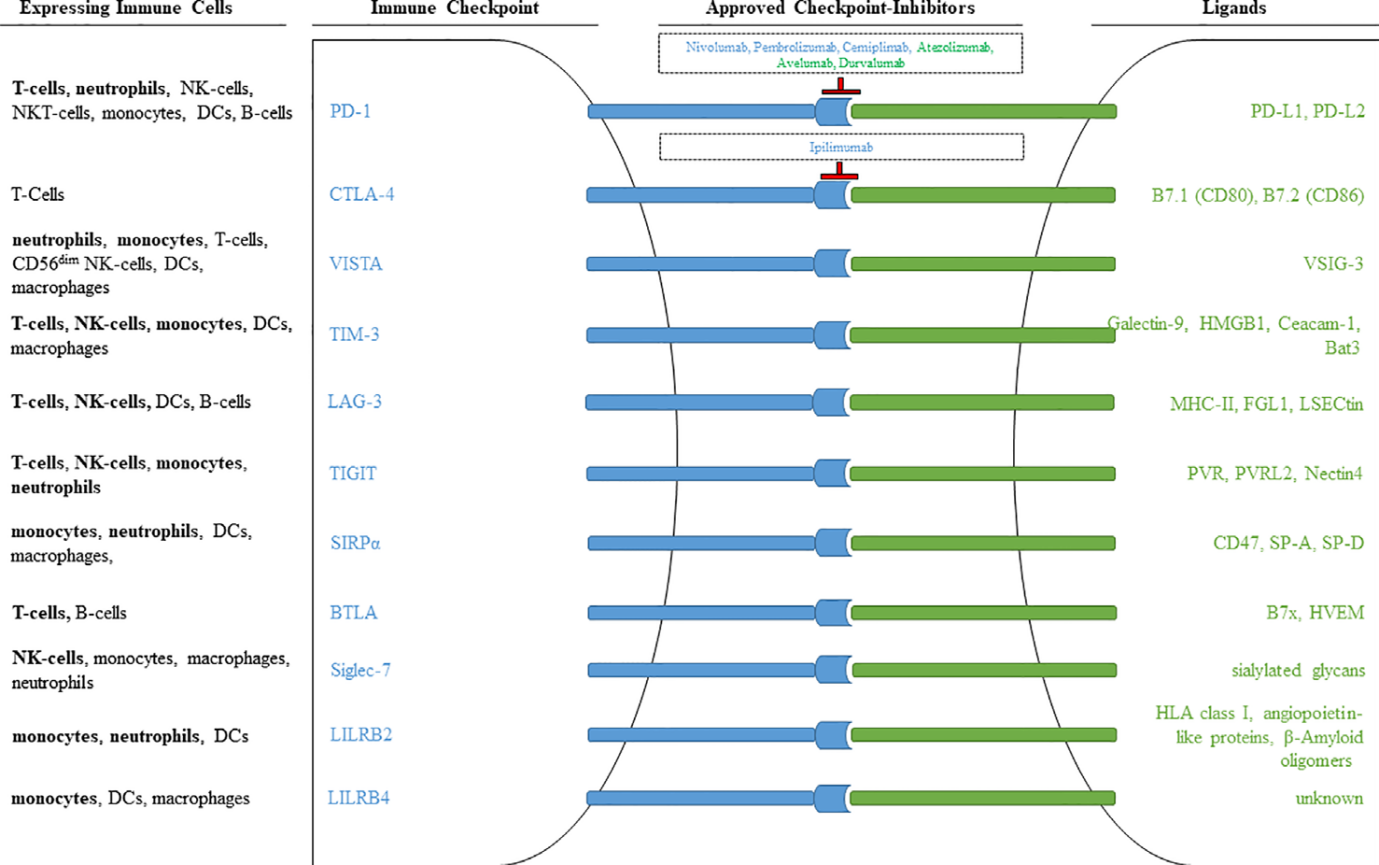
Immune checkpoints observed on different immune cells. Inhibitory receptors expressed on different immune cells are illustrated as blue rods, and ligands for these receptors are illustrated as green rods. FDA approved monoclonal antibodies that block receptor-ligand interaction are shown within the outlined boxes. Checkpoint inhibitors targeting the receptor are marked in blue, checkpoint inhibitors targeting ligands are marked in green. Immune cell populations printed in bold signalize that the respective immune checkpoint was included in our own antibody-panel (provided in **Table 1**) and that we were able to detect expression.

The described multiple functions of checkpoint molecules on cells of innate and acquired immunity not only allow to study the regulation of immune cells in detail, but also open new therapeutic possibilities. **Figure 13** shows essential checkpoint molecules, the expressing cells, and the ligands. Please note that there are only two of them target of approved therapies (PD-1 and CTLA-4).

### Tumors

#### VISTA

VISTA is a multipurpose immune regulator and therefore promising target for immunotherapy. Several studies observed the VISTA expression on various types of cancer cells and corresponding tumor infiltrating immune cells, e.g., in melanoma (221), gastric cancer (222), oral squamous cell carcinoma (223), pancreatic cancer (224) and pleural mesothelioma (225).

For instance, Gao et al. found elevated VISTA expression on peripheral blood monocytes in patients with metastatic prostate cancer receiving ipilimumab (anti CTLA-4 mAb) treatment suggesting VISTA’s inhibitory function may be relevant in advanced prostatic cancer (127). To show the inhibitory effect, they incubated monocytes untreated or pretreated with an anti-VISTA mAb with peripheral T-cells from patients. Untreated monocytes suppressed the IFN-γ production in peripheral T-cells whereas T-cells incubated with the pretreated monocytes showed normal IFN-γ production.

These results indicate that one way of VISTA carrying out its immunosuppressive function when expressed on monocytes is the inhibition of cytokine production in T-cells. Blocking VISTA may promote anti-tumor response and can be useful as a new therapeutic option for patients with metastatic prostate cancer.

Deng et al. assumed that VISTA expression may even be associated with reduced overall survival of cancer patients (128). They showed that VISTA upregulation on colon carcinoma samples correlated with a significant worse prognosis compared to low expressing samples. Further they demonstrated that tumor induced hypoxia leads to an increased VISTA expression on colon carcinoma cells and on tumor infiltrating leukocytes. Overexpression on monocyte derived suppressor cells (MDSC) contributes to T-cell suppression. Targeting VISTA expression on MDSC may be a useful therapeutic target to inhibit the MDSC mediated suppressive function, enhancing the immune response in patients with colon carcinoma.

To date, there are two phase one clinical trials (ClinicalTrials.gov: NCT02671955, NCT04475523) analyzing safety, pharmacokinetics, and pharmacodynamics of two different anti-VISTA monoclonal antibodies in advanced cancer patients.

#### TIM-3

Several studies showed the influence and importance of TIM-3 on immune response regulation in various cancers. According to Wang et al., TIM-3 expression on monocytes might be relevant for tumor progression in gastric cancer patients (226). They found increased TIM-3 expression on monocytes from gastric cancer patients. Elevated TIM-3 expression was associated with increased tumor depth and lymph node metastasis, indicating that TIM-3 expressing monocytes reduce the anti-tumor response and promote tumor growth and spread.

Circulating and tumor infiltrating NK-cells from patients with esophageal cancer express increased levels of TIM-3, with expression being higher on the CD56^bright^, than the CD56^dim^ subset. TIM-3 positive cells showed functional defects like decreased cytotoxicity and reduced production of IFN-γ and granzyme B. TIM-3 expression also correlated with lymph node metastasis, clinical stage, and tumor invasion (136).

Similar observations were made in patients with gastric cancer (137) and advanced melanoma (138), in which patients showed increased TIM-3 expression on peripheral blood NK-cells that correlated with poor prognostic factors. Blocking TIM-3 on the surface of NK-cells isolated from melanoma patients resulted in the internalization of the checkpoint molecule, upregulation of the IL-2 receptor (IL-2R) and most importantly an increased cytotoxicity and cytokine production (138).

Patients with lung adenocarcinoma also show higher TIM-3 expression, either when comparing their entire circulating NK-cell population or just the CD56^dim^ subpopulation individually to those of healthy donors. The CD56^bright^ subset appears to be unaffected. Overexpression on the mature NK cell subset correlated with bigger tumor size (≥ 3cm), higher tumor stage (T3-4), incidence of lymph node metastasis and shorter overall survival. Use of blocking antibodies against TIM-3 resulted in increased IFN-γ production and cytotoxicity by isolated NK-cells from patients against the human lung adenocarcinoma cell line A549 (227).

Furthermore, intratumoral NK-cells from patients with different cancers (i.e., colorectal, melanoma, bladder cancer) co-express TIM-3 and PD-1 to a higher extend than NK-cells in normal tissue from the same donor. Those TIM-3^+^ PD-1^+^ NK-cells appear to be exhausted based on their reduced ability to kill K562-target cells and to produce granzyme B and IFN-γ. Treatment with IL-21 can restore those effector functions *in vitro*. Moreover, injection of IL-21 into MHC-class I deficient tumors of Rag 1 -/- mice, led to an increase in tumor infiltration by NK-cells showing higher levels of IFN-γ and CD107a as well as reduced expression of TIM-3 and PD-1. Tumor growth was thereby inhibited (139).

Elevated TIM-3 expression can also be found on dendritic cells in the tumor microenvironment compared to normal environments. On tumor associated dendritic cells, TIM-3 suppresses inborn pattern recognition receptor mediated immune responses to nucleic acids. HMGB1 mediated activation of TIM-3 blocks the transport of nucleic acids into endosomal vesicles and thereby reduces the sensing system of nucleic acid (228).

#### LAG-3

In both pleural and peritoneal effusions of patients with malignant pleural mesothelioma, LAG-3^+^ NK-cells can be found, but the expressions vary strongly between patients (1.0 –68.1% LAG-3^+^ NK-cells of all NK-cells) (229). Further studies are needed to evaluate the role of LAG-3 expression in this context and other malignancies.

#### TIGIT

Reports about the TIGIT expression on NK-cells in patients with malignant diseases are indecisive. Increased expression of TIGIT on NK-cells in the peripheral blood has been reported in patients with myelodysplastic syndrome (230), high risk non-muscle invasive bladder cancer (231) and gastrointestinal cancer (gastric and colon cancer) (171). Patients with colon cancer show higher TIGIT expression on NK-cells in intratumoral regions than in peritumoral regions (232). On the other hand, there are reports that TIGIT expression on circulating NK-cells (cNK) does not change in patients with other neoplastic diseases such as AML (233), pancreatic cancer (234) and hepatocellular carcinoma (235). Interestingly, Chauvin et al. reported that TIGIT expression on circulating NK cells (cNK’s) of patients with melanoma did not differ from expression in healthy donors; only to later elaborate that TIGIT expression on tumor infiltrating NK-cells (TiNKs) in those patients is downregulated when compared to TIGIT expression on cNKs from both patients and healthy individuals. According to them, membrane bound CD155 can mediate internalization of TIGIT but not degradation (236).

Different tumor models in mice showed that TiNK-cells overexpress TIGIT which is accompanied by an exhausted phenotype. Treatment with anti-TIGIT mAbs resulted in an increased infiltration of active NK-cells into the intratumoral region, a reversion of the exhausted state (measured by increased expression of CD107a, TNF, IFN-γ, and CD226), inhibited tumor growth, reduced tumor metastasis and ultimately increased overall survival of the mice. Those effects were NK-cell dependent and did not rely on the presence of a functioning adaptive immune system (232). However, others reported that application of anti-TIGIT mAbs only reduced metastasis when combined with IL15/IL15R treatment in their tumor bearing mice models (236). Right now there are multiple clinical trials registered, that investigate both safety and efficacy of anti-TIGIT mAbs in the treatment of a variety of malignant diseases (e.g., NCT04047862, NCT04353830, NCT02964013, NCT04543617, NCT4732494, NCT04732494, NCT04693234).

#### SIRPα

Various cancer types including solid tumors as well as hematological malignancies have shown to harness the SIRPα/CD47 pathway to evade immune surveillance by overexpressing CD47. To name a few: acute lymphoblastic leukemia (237), non-Hodgkin’s lymphoma (NHL) (238), multiple myeloma (239), B-cell lymphoma (240), leiomyosarcoma (241), breast cancer (242) and osteosarcoma (243).

In this context Seifert et al. analyzed the SIRPα expression on cells from patients with primary myeloid leukemias (55). Immature leukemic blasts showed no or significantly reduced SIRPα expression suggesting the possibility that reduced SIRPα expression is a cause or consequence of aberrant proliferation of these cells.

SIRPα expression is not only limited to tumor cells but also expressed on tumor infiltrating immune cells. Cabrales et al. showed that SIRPα expression on monocytes may play a role in cancer (244). They studied the effects of RRx-001, an anti-cancer agent used in clinical trials, on tumor cells and monocytes. RRx-001 reduced SIRPα expression *in vitro* and thus constrained the CD47-SIRPα signaling axis which ultimately enhanced both immune response and phagocytosis as well as antigen processing and presentation. RRx-001 also promoted the switch from M2 to M1 macrophages in the tumor microenvironment promoting M1-mediated proinflammatory antitumor conditions.

In patients with NHL, there may be differentiated between three monocyte subsets according to their SIRPα expression: CD14^+^SIRPα^high^, CD14^-^SIRPα^low^ and CD14^-^SIRPα^neg^. To analyze the impact on T-cell activation Chen et al. cultured T-cells with these three monocyte subsets finding out that T-cell proliferation was inhibited by monocytes expressing SIRPα at high and low levels but not by monocytes that are SIRPα^neg^ (245).

When comparing the phagocytic function of these three subsets, the authors demonstrated that CD14^+^SIRPα^high^ monocytes showed the strongest increase in phagocytic activity after blocking SIRPα with an Fc fragment. The activity in CD14^-^SIRPα^low^ and CD14^-^SIRPα^-^ monocytes was lower but also enhanced. The SIRPα-Fc downregulated even CD47 on monocytic surfaces confirming the reduced signaling *via* the CD47-SIRPα axis. Blocking the CD47-SIRPα pathway may result in enhancement of immune activity and phagocytosis rate. Therefore, SIRPα expressing phenotypes may have better clinical prognosis due to new therapeutic possibilities.

So far, immunotherapy exploiting checkpoint inhibition has focused on targeting the adaptive immune system, especially T-cells. Targeting CD47 respectively SIRPα and therefore targeting the innate immune system provides a novel approach in cancer therapy. As described in the examples above, this approach may be promising. Currently, there are multiple preclinical and clinical trials testing biosafety, tumor specificity and effectiveness of anti-CD47 antibodies, anti-SIRPα antibodies and SIRPα-Fc fusion proteins [reviewed in (246)].

#### BTLA

Upregulation of BTLA is important for restricting the expansion and function of NY-ESO-1 (New York esophageal squamous cell carcinoma-1) specific CD8^+^ T-cells in melanoma. BTLA^+^ PD-1^+^TIM-3^-^ CD8^+^ T-cells are the largest group of NY-ESO-1–specific CD8^+^ T-cells. These cells are partially dysfunctional producing less IFN-γ than BTLA^-^T-cells. T-cells expressing all three immune checkpoints PD-1, TIM-3 and BTLA are highly dysfunctional and produce less IFN-γ, TNF-α and IL-2. In contrast to the negative correlation between T-cell functionality an PD-1 expression, BTLA expression remains constant showing no further increase. This leads to the assumption that a higher BTLA expression is rather independent of functional exhaustion and powered by high antigen load. In addition to PD-1 and TIM-3 blockade, BTLA blockade enhances the NY-ESO-1-specific CD8^+^ T-cells functions (247) and is a promising therapeutic option for NY-ESO-1 patients.

#### Siglec-7

Tao et al. analyzed NK cells in patients with hepatocellular carcinoma showing a reduced number of NK-cells and decreased proportion of the mature NK cell subset. Among the circulating NK-cells, the frequency of Siglec-7 expression is significantly decreased, regardless of whether a patient is positive or negative for HBV or HCV infection (235).

Further studies on patients with other cancer entities showed normal expression levels. The frequency of Siglec-7^+^ circulating NK-cells in patients with colon adenocarcinoma and malignant melanoma are similar to healthy individuals (204). Regulation of transcription appears to be the main factor for the level of Siglec-7 expression. Hypomethylation of CpG site 8 and 9 within a CpG island in the 5’ Siglec-7 promotor increases Siglec-7 surface expression. Furthermore, histone modification through the use of histone deacetylase inhibitors also results in higher Siglec-7 surface levels. DNA methyltransferase inhibitors and histone deacetylase inhibitors are used to fight leukemia but it is currently unknown if or how changes in the expression of Siglec-7 on NK-cells contribute to the effects of this course of treatment (248).

#### LILRB

Another potentially important checkpoint in cancers is LILRB2. Sun et al. describe the expression on non-small-cell lung carcinoma (NSCLC) and show the correlation between high LILRB2 expression and reduced infiltration of lymphoid cells in the tumor tissue. This confirms the inhibitory effect of LILRB2 due to reducing lymphocytic immune response (249).

Similar results were found by Liu et al. (250). LILRB2 is overexpressed on lung tissue from patients with lung carcinoma in comparison with normal lung tissue that did not express the receptor. A549, a NSCLC cell line, showing the highest expression, was used for their further experiments. Using shRNAs to inhibit LILRB2 expression, they demonstrated that the cultured A549 cancer cells were significantly slower in proliferation and had an increased cell death suggesting that LILRB2 overexpression enhances tumor growth (250).

Further LILRB 4, and also LILRB1 expression is detected on gastric cancer cell lines. Less differentiated cell lines show higher expression compared to differentiated cell lines. To compare the cytotoxicity of NK-cells in a LILRB1^low^LILRB4^low^ (high differentiated) gastric cancer cell line with a LILRB1^high^LILRB4^high^ (low differentiated) gastric cell line, the gastric cancer cell lines were co-cultured with the natural killer cell line NK92MI showing reduced NK cytotoxicity in the poorer differentiated gastric cancer cell line. This leads to the suggestion that LILRB4 and 1 expression correlate with poor differentiation of gastric cancers and effectively suppress NK-cell activity (251).

LILRB4 overexpression is also detected on pancreatic cancer (252) and breast cancer (253) cells.

Elevated LILRB expression is not limited to solid cancer cells but also found in hematological malignancies such as AML. Especially cells of patients with AML M4/5 monocyte differentiation have a significantly higher LILRB4 expression compared to other forms of AML. LILRB4 expression is more sensitive and specific for AML M4/5 than other differentiation markers used in flow cytometry and can be used as a diagnostic marker (254).

The importance of LILRB4 expression in therapy of AML patients is described by John et al. (255). One promising treatment option for AML patients are CAR-T-cells. Unfortunately, therapy is limited due to the lack of an AML blast specific antigen and occurring side effects such as myelotoxicity and – suppression. Since LILRB4 is specifically expressed by nearly all monocytic AML subtype M5 cells, John et al. developed an anti-LILRB4 CAR transducing it into T-cells. Using a mouse model, they demonstrated the efficiency of these T-cells on fighting leukemic blasts compared to an untreated control group. LILRB4 expression is not found on hematopoietic stem cells or pluripotent progenitor cells. Therefore, side effects occurring in the common CAR-T-cell therapy are not expected making LILRB4-CAR-T-cells a new efficient therapeutic option for patients with AML.

LILRB 2 and 4 as negative immune checkpoint molecules being expressed on hematological and solid tumors downregulating innate and adaptive immune response may be relevant therapeutic approaches and targets in anti-tumor treatment. Blocking LILRB expression with an antibody or altering their signal transduction with a specific high-affinity ligand could enhance an anti-tumor immune response and inhibit tumor growth (209). Further studies are needed to prove these effects and therapeutic targets need to be evaluated in clinical trials.

### Infection

#### PD-1

Sepsis is a life-threatening disease due to a dysregulated and excessive immune response. Xia et al. analyzed the effect and expression of PD-1 on monocytes in septic patients using flow cytometry (109). They showed that in septic patients CD14^+^CD16^+^ monocytes have a significantly increased PD-1 expression compared to healthy controls. When blocking PD-1 with an antibody and stimulating the cells with LPS, the proportion of pro- and anti-inflammatory cytokines TNF-α- and IL-10-secreting monocytes increased. These results suggest that PD-1 may dysregulate monocyte function in septic patients, especially the inflammatory CD14^+^CD16^+^ monocyte subset. Blocking the PD-1 pathway may enhance the secretory function of monocytes which is important for balancing the immune response.

PD-1 expression is also found on monocytes of septic neonates. Zasada et al. described the expression on the different monocyte subsets in preterm neonates with late-onset sepsis (LOS) (256). They showed that neonates with LOS had an increased number of all monocyte subsets. The percentage and number of classical and intermediate monocytes expressing PD-1 was elevated. Neonates with LOS who developed a septic shock had an increased number of intermediate monocytes and the percentage and number of intermediate monocytes expressing PD-1 were significantly elevated compared to neonates without a septic shock. PD-1 expression may be an important factor regulating immune responses and a potential therapeutic target to possibly improve outcome in septic patients.

Similar results were shown for patients with Q-fever endocarditis. PD-1 was also upregulated on the intermediate monocyte subset in patients with Q-fever. When incubating monocytes with *C. burnetii*, the gram-negative bacterium causing Q-fever, PD-1 upregulation was detected. Further investigation on PD-1 modulation with LPS from E. coli also showed an increased PD-1 expression on monocytes compared to unstimulated cells (107).

PD-1 upregulation is also seen on all monocytes subsets in patients with HIV compared to healthy controls. In acute HIV infection and chronic HIV infection without antiretroviral therapy, especially the intermediate subsets showed an elevated expression of PD-1 compared to treated patients. The non-classical monocytes showed an elevated PD-1 expression mainly in chronic untreated patients compared to acute and chronic treated infection. PD-1 expression on both subsets correlates positive with the frequency of regulatory, also called suppressor, T-cells suggesting that elevated PD-1 expression on monocytes promotes T-cell exhaustion and downregulation of immune response in patients with HIV (110).

Herpes simplex virus 1, a chronic infection, causes exhaustion in antiviral T-cells. HSV-specific CD8^+^ T-cells have a higher expression of PD-1 and LAG-3 receptors in symptomatic patients with a recurrent herpetic disease than in asymptomatic patients. A combined blockade of LAG-3 and PD-1 pathways improved the function of antiviral CD8^+^ T-cells in the cornea and the trigeminal ganglia of rabbits (257).

#### VISTA

The immunosuppressive function of VISTA can be beneficial in autoimmune diseases to decrease inflammation and disease activity. Bharaj et al. found out that VISTA is up regulated on monocytes of HIV-infected individuals, especially on the intermediate inflammatory subset (CD14^+^CD16^+^), which induce secretion of high levels of proinflammatory cytokines (126). Furthermore, this overexpression stimulated T-cells from HIV-seropositive individuals and, in contrast, blocking VISTA on monocytes reduced T-cell induced cytokine production in these individuals. In HIV the activation of the immune system negatively influences the course of the disease and VISTA expression on monocytes correlates with this activation. Blocking VISTA expression on monocytes could be a new therapeutic approach.

There are several factors that modulate VISTA expression on monocytes. Bharaj, et al. described the influence of several TLR agonists and cytokines (126). Poly : IC (TLR3) and Flaggelin (TLR5) induced an upregulation suggesting that VISTA might be increased during viral and bacterial infections. Also, significant upregulation was induced by IL-10 and INF-γ. No effect was seen after stimulation with TLR4 (LPS). TLR8/9 ligands caused a downregulation.

#### TIM-3

It has been shown that the expression of TIM-3 is increased in HIV-1 infected individuals in comparison to uninfected individuals. There is a positive correlation between the TIM-3 expression and the HIV-1 viral load. HIV-1 –specific CD8^+^ T cells showed an upregulated expression of TIM-3. T-cells with TIM-3 expression did neither produce cytokines nor showed proliferation in response to the antigen. The proliferation and cytokine production could be restored by blocking the signal pathway of TIM-3 in HIV-1 specific T-cells (258). CD56^bright^ but not CD56^dim^ NK-cells from untreated HIV patients show higher TIM-3 levels than a healthy control group. After 6 months of combined antiretroviral treatment this overexpression is reverted to normal (259).

Similar findings were described in Hepatitis C infected patients. There is an increased expression of TIM-3 on CD4^+^ and CD8^+^ T-cells in individuals with chronic hepatitis C infection. A high expression of TIM-3 correlates with dysfunction and reduced cytokine production, which can be restored by blocking the TIM-3 pathway (260).

Hepatitis C virus (HCV) infection also causes increased TIM-3 expression on CD56^dim^ but not on CD56^bright^ NK-cells (261). Transcription factor T-bet is also up-regulated in NK-cells from HCV patients. Furthermore, miR-155 is decreased by tenfold. Reconstitution of this micro-RNA results in a reduction of both T-bet and TIM-3 expression (262). TIM-3^high^ NK-cells from HCV patients do not only show an activated phenotype (higher expression of activating receptors NKp30, NKp46, NKG2C, NKG2D, lower expression of inhibitory receptor NKG2A) but also a greater ability to kill target cells upon pre-activation with lymphokines. They are also better at inducing the expression of TRAIL upon IFN-α stimulation and at controlling HCV in an *in-vitro* model. Cytokine production was comparable to TIM-3^low^ NK-cells. TIM-3 expression remained high even when IFN-α based antiviral therapy successfully led to viral eradication (261). Treatment of NK-cells from HCV patients with anti-TIM-3 antibodies resulted in increased IFN-γ expression. Given that the blockade also enhanced phosphorylation of STAT-5, it can be speculated whether TIM functions through interference with the Jak/STAT pathway within NK-cells (262).

Wang et al. evaluated the role of TIM-3 on monocytes in patients with chronic Hepatitis C receiving recommended Hepatitis B vaccination (263). They revealed that TIM-3 was overexpressed on monocytes in Hepatitis B vaccine non-responders. First, they examined IL-12 and -23 production in monocytes after LPS stimulation in patients with chronic hepatitis finding out that cytokine production in patients with chronic HCV is reduced compared to healthy controls. When comparing vaccine responders and non-responders, similar results were shown; non-responders had reduced cytokine levels. To show that TIM-3 expression may be responsible for this inhibitory effect on monocytes, TIM-3 expression was examined with flow cytometry. Same result as for cytokine production was obtained meaning chronic HCV patients and non-vaccine responders had elevated TIM-3 levels. These results suggest that TIM-3 expression may downregulate IL-12 and -23 expression. Using a TIM-3 mAb proved this suggestion because cytokine production in monocytes increased after TIM-3 blockade and stimulation with LPS (263). These results show TIM-3’s potential influence on vaccine response.

Circulating NK-cells from patients with a chronic hepatitis B virus infection also show higher expression of TIM-3 than their counterparts in healthy donors. This overexpression is weakly correlated with higher levels of alanin transaminase, which can be an indicator of a bad prognosis. In an ex-vivo model anti-TIM mAb’s were able to significantly improve the cytotoxicity of NK-cells isolated from chronic hepatitis B patients towards Hep2.2.15 cells (264).

#### LAG-3

LAG-3, PD-1 and TIGIT are immune checkpoint molecules which are positively associated with the frequency of CD4^+^ T-cells with HIV DNA. CD4^+^ T-cells with all 3 checkpoints expressed are highly enriched for integrated viral genomes. Most of the T-cells with at least one of these checkpoints carried HIV genome. To target latently infected cells in HIV suppressed individuals, immune checkpoint blockers against LAG-3, PD-1 and TIGIT could be a valuable option (265). High expression levels of immune checkpoints such as LAG-3, PD-1, TIM-3 and CD38 on CD8^+^ T-cells show a correlation with T-cell exhaustion and increased clinical disease progression as well as duration of infection (266).

HIV positive women who had received antiretroviral therapy (ART) show significantly higher frequencies of LAG-3^+^ NK-cells than HIV negative women. The expression of the checkpoint molecules did not correlate with CD4 count, CD4 recovery or ART duration (267). Taborda et al. also reported that HIV progressors express LAG-3 more frequently than HIV controllers (<2000 copies/ml for ≤1 year without ART) (268).

Merino et al. studied adaptive NK-cells in the context of human cytomegalovirus infection (269). Adaptive NK-cells show a certain pathogen specificity, long-term persistence, and control of secondary infection. Chronic stimulation of adaptive NK-cells results in a significant upregulation of LAG-3 and PD-1. Hypomethylation within the promotor regions of their gens appears to be responsible for the induction of both PD-1 and LAG-3. LAG-3 positive adaptive NK-cells produced less IFN-γ in response to stimulation with K562 cells compared to LAG-3 negative adaptive NK-cells but showed a comparable rate of degranulation.

#### SIRPα

Under pro-inflammatory stimuli like LPS or TNF-α, Londino et al. demonstrated that SIRPα proteolysis is enhanced (270), abrogating its inhibitory function which results in enhancement of inflammatory signaling *via* JAK/STAT pathway. This leads to activation of the immune response. This result suggests that SIRPα may play an important role in regulating inflammatory conditions due to lack of its inhibitory function.

Smith et al. analyzed the role of SIPRα on regulating the innate immune response towards different pathogens like gram^+^ or gram^-^ bacteria or yeast (187). Incubation with a murine anti-human SIPRα mAb and stimulation with LPS resulted in reduced production of proinflammatory cytokine TNF-α but had no effect on other cytokines. Similar results were found when LPS was replaced by zymosan or mycobacterial antigens. These result show that SIPRα inhibits the immune response under inflammatory conditions.

The importance of SIPRα regulating the monocyte response during inflammation was also shown by Liu et al. (271). They demonstrated that SIRPα reduces β2-integrin-mediated monocyte adhesion, transendothelial migration, and phagocytosis. Thus, it may serve as a critical molecule in preventing excessive activation.

Therefore, they created SIRPα overexpressing THP-1 cells. SIRPα significantly reduced the upregulation of surface b2-integrin by chemokine MCP-1. β2-Integrin is responsible for adhesion to endothelial cells. With the help of a transmigration assay, transendothelial migration on SIRPα overexpressing cells was analyzed showing a reduced migration of monocytes in the presence of MCP-1, which was even further reduced in the absence of MCP-1. The same was shown for phagocytosis. SIRPα overexpressing cells showed decreased phagocytosis of fluorescein-labeled E. coli compared to mock-transfected cells. All these results indicate that SIRPα is important for regulating monocyte and macrophage responses. Nevertheless, this downregulation may be important in some diseases such as early stage of arteriosclerosis where monocytes contribute to disease progression. In this case SIRPα overexpression would be beneficial (271).

#### Siglec-7

Varchetta et al. showed that untreated patients with HCV or HBV virus possess a lower frequency of circulating Siglec-7^+^ NK-cells than healthy donors (272). Meanwhile they were able to detect increased serum levels of Siglec-7. The expression among HCV patients is inversely correlated with negative indicators of disease progression like liver cell injury, liver stiffness, fibrosis scores and histological fibrosis. Higher frequency of Siglec-7^+^ NK-cells at baseline is also a positive predictor of sustained virological response after treatment with IFN-α and ribavirin.

Even though HIV-1 is not able to directly infect NK-cells, it is able to impair their cytolytic function and induce phenotypical changes. During the first response of the innate immune system in the early stages of infection, patients show an increasing subset of Siglec-7^-^/CD56^+^ NK-cells. This subset shows reduced degranulation and cytokine production. The loss of Siglec-7 is dependent on ongoing viral replication since this change cannot be observed in long-term non-progressors. A suppression of the virus with ART to undetectable levels can revert the loss of Siglec-7 expression (273).

#### LILRB

In inflammatory conditions LILRB expression can be upregulated. Brown et al. analyzed LILRB2 and 4 expression on salmonella infected APCs (274). Macrophages showed an LILRB2 and 4 upregulation during Salmonella infection regardless of whether heat killed or viable Salmonella typhimurium bacteria were used. Other TLR-ligands like LPS and flagellin also induced higher expression, though flagellin not as strong as the other ligands. Furthermore, macrophages had an altered, but statistically not significant, cytokine secretion with increased anti-inflammatory cytokine IL-10 and decreased pro-inflammatory IL-8. Upregulation of LILRB during infection could be a regulatory mechanism by the immune system to prevent excessive damage and reduce inflammation.

Venet et al. analyzed the LILRB2 expression on patients with septic shock. In comparison with healthy donors LILRB2 expression was generally increased on monocytes and higher on the nonclassical CD16^+^ subset. These results propose that elevated LILRB2 expression on monocytes in septic shock patients may play a role in altered immune response in patients with sepsis. These findings could be confirmed under inflammatory conditions ex vivo (214).

Baffari et al. investigated the cause of LILRB2 upregulation on monocytes in septic patients. They found out that there was an association of organ dysfunction in septic patients and LILRB2 surface expression on monocytes. Patients with severe dysfunctions had elevated checkpoint molecule levels. They incubated blood from healthy donors with sera from septic patients where an upregulation of LILRB2 on monocytes could be seen. This suggest that factors in the serum of septic patients may be responsible for the increased checkpoint expression leading to a more severe condition. Furthermore, they pointed out that immunosuppression caused by LILRB2 may have a negative influence on mortality and morbidity in septic patients. On the other hand, this inhibition may prevent an uncontrolled excessive immune response that would worsen the condition (275).

LILRB2 expression on monocytes of patients with HIV (AIDS, acquired immune deficiency syndrome) was analyzed by Vlad et al. They found LILRB2 upregulation on the monocytic surface and a switch into a more anti-inflammatory phenotype indicated by an altered cytokine secretion. Blood from healthy donors incubated with HIV patients’ sera lead to an increase of LILRB2 expression on monocytes as well. This suggests that HIV infection alters function of antigen-presenting cells by upregulating the inhibitory checkpoint LILRB2 and by increased secretion of anti-inflammatory cytokines (276).

### Autoimmunity

#### CTLA-4

**CTLA-4** on T_reg_ is important to prevent autoimmunity and controls the activity of other cells such as APCs and naïve T-cells (277, 278). Its expression on activated T-cells regulates T-cell activation by reducing IL-2 production and also IL-2 receptor expression (92, 279). Both may be important for therapies aiming for specific immunosuppression in autoimmune diseases and for transplantation settings. Immunosuppressants are for example the CTLA-4 fusion protein Belatacept, which binds B7 and thereby prevents co-stimulation by CD28. Another one is Abatacept, this fusion protein is commonly used in treatment of rheumatoid arthritis.

#### VISTA

Studies with murine models have shown that **VISTA** deficiency is accompanied by a higher risk for autoimmune disease (121, 280–282).

Ceeraz et al. had a closer look on the impacts of VISTA on a murine model of lupus (129). They examined the VISTA expression by flow cytometry in Sle1. Sle3 lupus prone mice in comparison with B6 mice used as controls. They showed that VISTA expression in the inflammatory monocyte compartment is reduced during active lupus assuming that VISTA deficiency might lead to an increased disease activity. Blocking VISTA with a mAb would enhance the disease. They also showed that myeloid cells of VISTA deficient Sle1.Sle3 mice had a heightened activation status that correlated with increased cytokine production. Their data demonstrated the importance of VISTA in regulating autoimmune disease and in this model preventing disease progression (129).

Wang et al. describe similar results. In experimental autoimmune encephalomyelitis which is a murine disease model for human multiple sclerosis, anti-VISTA treatment provoked disease exacerbation (54).

#### TIGIT

Kurita et al. examined the frequency of TIGIT expression on CD4^+^ T-cells in patients with atopic dermatitis and found a higher expression compared to a healthy control group. They stated that this could indicate that TIGIT may function as a partial inhibitor to autoimmune reactions and skin inflammation. They also discussed the possibility that a lower expression of TIGIT in certain patients may lead to an exacerbated activity of atopic dermatitis (283).

#### LILRB2

LILRB2 as a negative immune checkpoint molecule may be relevant in neurological diseases such as Alzheimer disease (284). Kim et al. showed in a study with human transgenic mice that the mice LILRB2 homologue PirB can bind β-Amyloid oligomers. This binding engages colfilin, a PirB ligand, responsible for actin depolymerization resulting in synaptic loss and “altered synaptic plasticity and cognitive deficits”. Similar mechanisms are suggested in patients with Alzheimer disease. Blocking LILRB2 may be a beneficial therapeutic approach to reduce the neuronal damage and therefore disease progression.

In rheumatoid arthritis, LILRB2 expression is found on immune cells in the synovial tissue. Huynh et al. suggested that LILRB2 expression and function may be altered under disease modifying antirheumatic drugs (DMARD’s) (285). They treated macrophages differentiated from THP-1 with dexamethasone, methotrexate and cyclosporine A and stained them with anti-LILRB2 mAbs. Patients responding to treatment showed a reduced number of inflammatory cells and reduced LILRB2 expression on tissue macrophages, compared to non-responders who showed increased number and expression.

Chang et al. examined the LILRB2 expression on monocytes incubated with CD8^+^ T-cells (220). Flow cytometry analysis showed upregulation of LILRB2 expression and downregulation of co-stimulatory receptors such as CD86. To determine the role of this upregulation they evaluated the LILRB2 expression in patients with heart transplantation. CD8^+^ T-cells from these patients were isolated and incubated with monocytes from a control individual. They revealed that patients without acute rejection within the first 6 months showed an upregulation of LILRB2 which was not the case in patients with acute rejection. This suggests that CD8^+^ T-cells induce a tolerogenic phenotype in monocytes characterized by LILRB2 upregulation that reduces immune responses after transplantation and supports acceptance of the donated organ.

#### LILRB4

One study demonstrated that LILRB4 expression on monocytes in patients with multiple sclerosis can be upregulated upon stimulation with Vitamin D3 and IFN-γ. Combined stimulation had an additive effect (217). Vitamin D3 and IFN-γ could therefore be useful in patients with multiple sclerosis to reduce the cerebral inflammation in a LILRB4 dependent fashion.

## Conclusion

We are convinced that the new immunological tumor therapies and the rapidly growing knowledge about the importance of checkpoint molecules in malignant, infectious, and autoimmune diseases will generate a broad demand for appropriate flow cytometric assays. It is not expected that ready-to-use test kits will be available at an early stage. Here, we have placed next to the literature review a selection of flow cytometric examples of how, with appropriate effort, diagnostic laboratories can offer these examinations. In this way, it should be possible to meet this current challenge in immunodiagnostics.

## Author Contributions

Conceptualization, A-RB, UK, AB, and US. Methodology, AB and US. Resources, UK, AB, and US. Data curation, AB. Writing - original draft preparation, BS (NK-cells), MW and FF (T-cells), MKW (Monocytes), KF (PMNs), and SS (B-cells and DC). Writing – BS, KF, A-RB, and US. Visualization, BS (NK-cells), MW and FF (T-cells), MKW (Monocytes), KF (PMNs), and AB. Supervision, UK, SF, and US. Project administration, US. All authors contributed to the article and approved the submitted version.

## Funding

The authors acknowledge support from the Leipzig University within the program of Open Access Publishing.

## Conflict of Interest

The authors declare that the research was conducted in the absence of any commercial or financial relationships that could be construed as a potential conflict of interest.
